# Supplementary figures and images for: Regulated bacterial interaction networks: A mathematical framework to describe competitive growth under inclusion of metabolite cross-feeding
Source: PLoS Comput Biol. 2023 Aug 21;19(8):e1011402. doi: 10.1371/journal.pcbi.1011402 (PMC10470959; doi:10.1371/journal.pcbi.1011402)

A

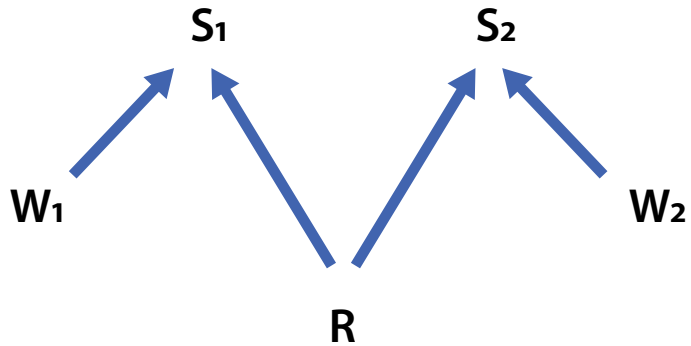

B

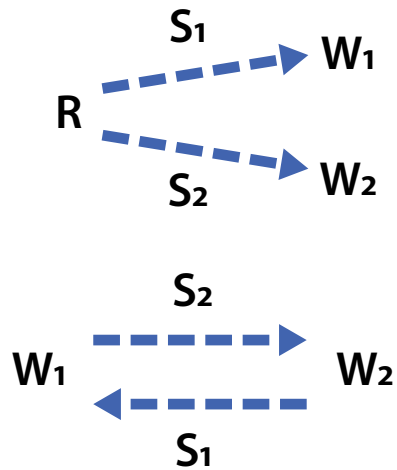

Supplement: S1 Fig — (A) The predator-prey Lotka-Volterra representation where species S1 preys on R and W2 and S2 preys on R and W1. (B) The catalytic conversion representation involving species R, W1 and W2. The species associated to the dashed arrows indicate the nature of the catalyst, and the direction of the arrow indicates the transformation product. (PDF) [file pcbi.1011402.s001.pdf]

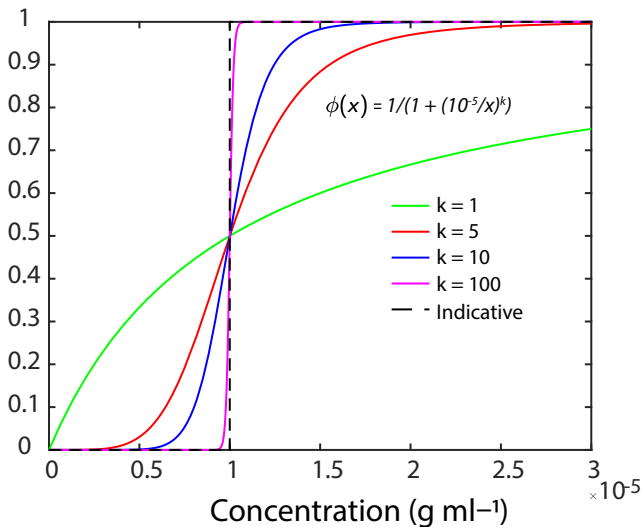

Supplement: S2 Fig — Figure shows activation function outcome dependent of the waste concentration for different k-values of the Hill function, and in comparison to the indicative (step) function. (PDF) [file pcbi.1011402.s002.pdf]

**A**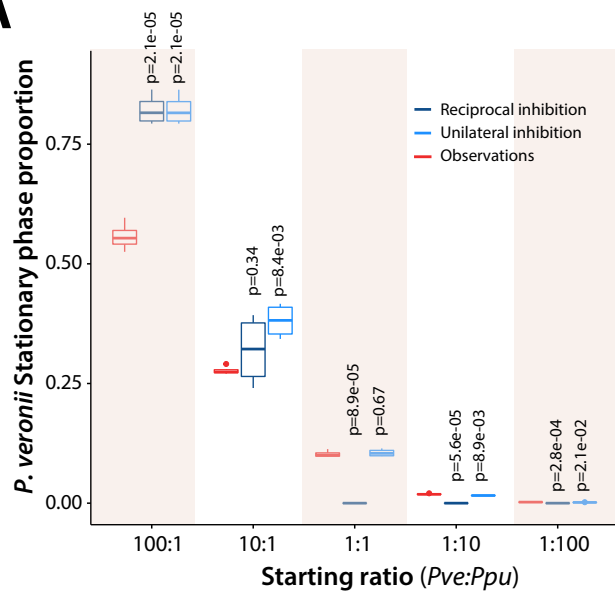**B**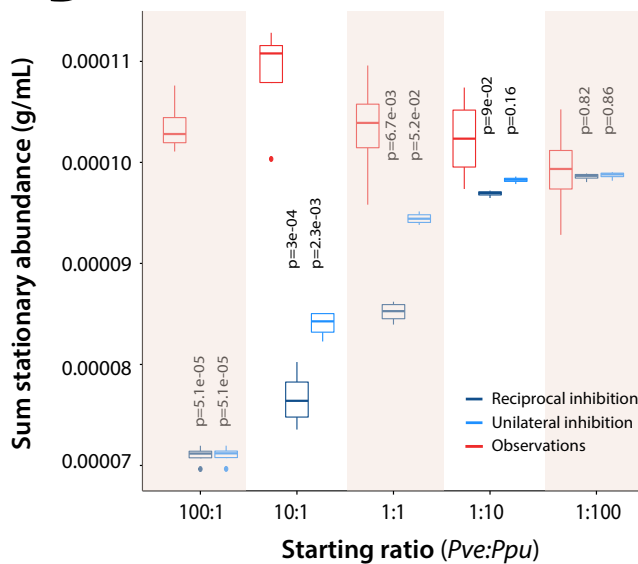**C****Reciprocal inhibition**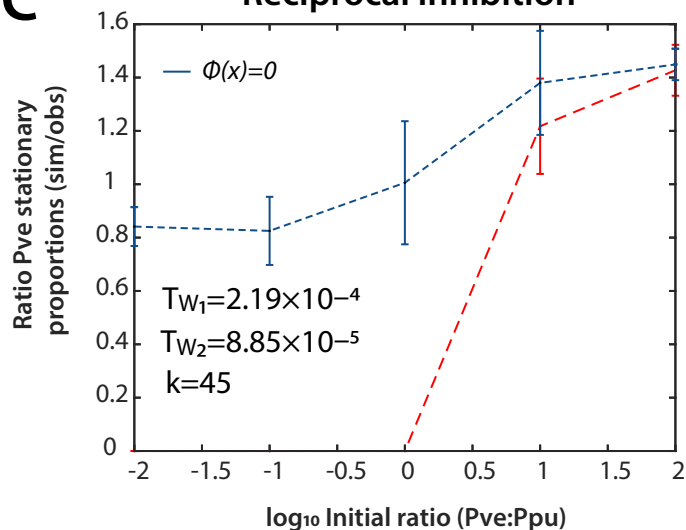**D****Unilateral inhibition**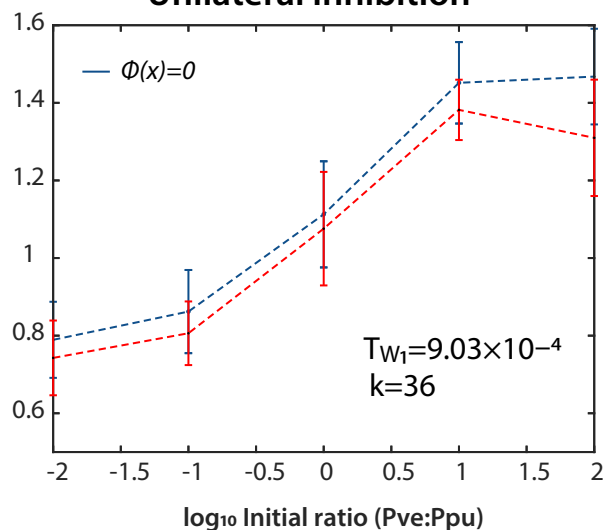

Supplement: S3 Fig — (A) Simulated versus empirical observed P. veronii stationary phase proportions at different starting cell ratios for the reciprocal and unilateral inhibition. (B) as A, but for the sum of co-culture biomass. (C) Effect on the ratio of simulated versus observed P. veronii steady-state proportions for the model without any assumed interactions (ϕ = 0, dark blue lines) and reciprocal interactions (red lines, n = 5 simulations). (D) as (C), but with unilateral inhibition. Used threshold values for the reciprocal inhibition are TW2=8.85e-05, TW1=2.19e-04 and Hill factor k = 45. For the unilateral inhibition simulation, we used TW1=9.03e-04 and a Hill factor of k = 36. (PDF) [file pcbi.1011402.s003.pdf]

A

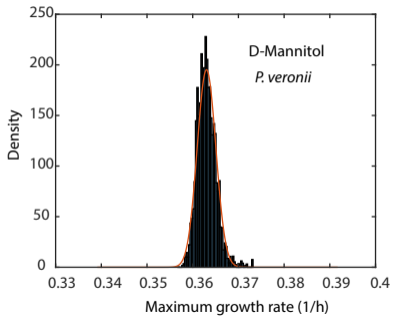

B

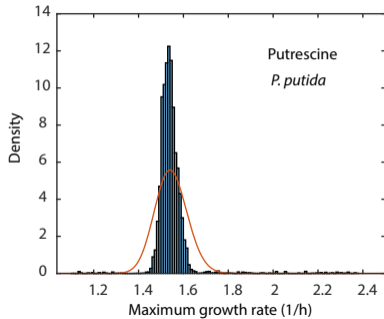

Supplement: S4 Fig — Plots show histograms of maximum growth rates inferred from Metropolis-Hasting fitting with the Markov Chain Monte Carlo approach, as described in the Materials and Methods section. (PDF) [file pcbi.1011402.s004.pdf]
