# Supplementary material for: Regulated bacterial interaction networks: A mathematical framework to describe competitive growth under inclusion of metabolite cross-feeding
Source: PLoS Comput Biol. 2023 Aug 21;19(8):e1011402. doi: 10.1371/journal.pcbi.1011402 (PMC10470959; doi:10.1371/journal.pcbi.1011402)
Supplement: S5 Fig — Plots show simulated biomass growth in 8 replicates of P. putida (blue) or P. veronii (green) in co-culture on a mixture of D-mannitol (red) and putrescine (brown), and predicted waste concentrations (light green and blue), for five different cell starting ratios (100:1, 10:1, 1:1, 1:10 and 1:100, as indicated), and with 1 × 10^6^ cells per ml at start. Simulations in (A) assume a Monod model without cross-feeding, whereas (B) includes cross-feeding using the discontinuous threshold function). (PDF) [file pcbi.1011402.s005.pdf]

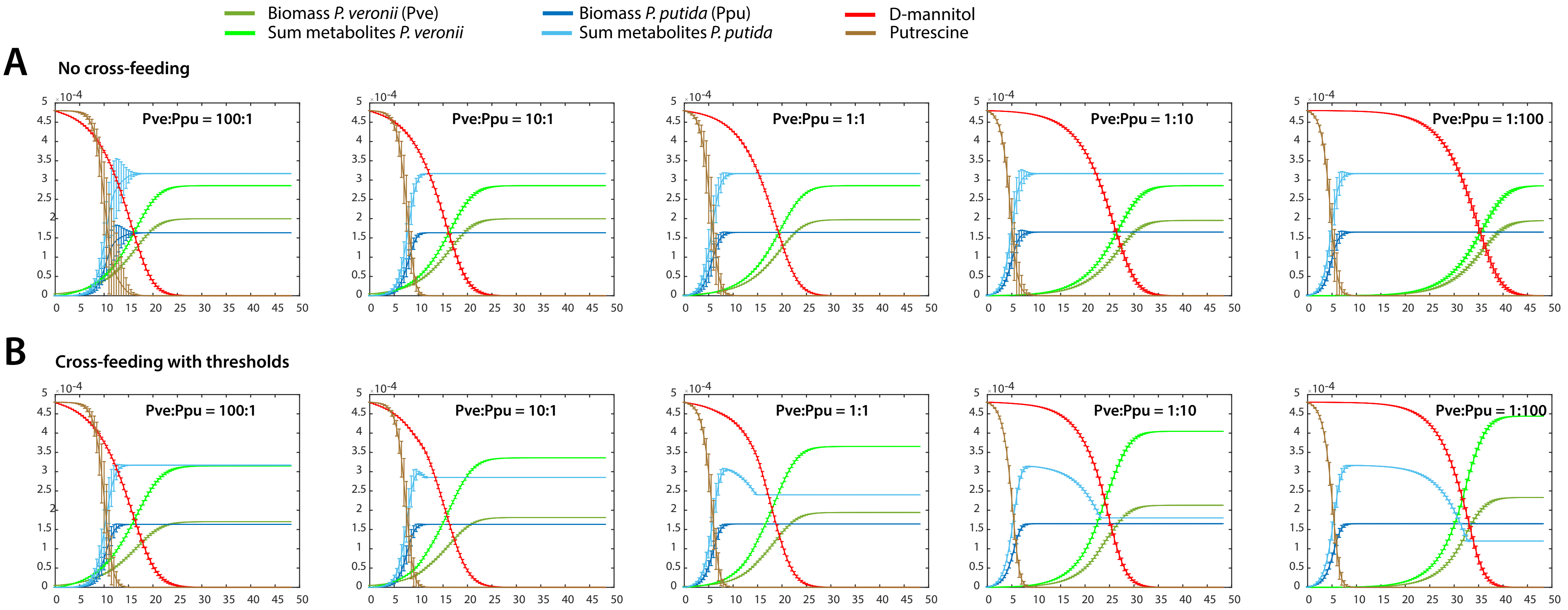

Biomass P. veronii (Pve)
Sum metabolites P. veronii
Biomass P. putida (Ppu)
Sum metabolites P. putida
D-mannitol
Putrescine
A
No cross-feeding
Pve:Ppu = 100:1
Pve:Ppu = 10:1
Pve:Ppu = 1:1
Pve:Ppu = 1:10
Pve:Ppu = 1:100
B
Cross-feeding with thresholds
Pve:Ppu = 100:1
Pve:Ppu = 10:1
Pve:Ppu = 1:1
Pve:Ppu = 1:10
Pve:Ppu = 1:100
×10-4
0 0.5 1 1.5 2 2.5 3 3.5 4 4.5 5
0 5 10 15 20 25 30 35 40 45 50
