# Supplementary material for: Regulated bacterial interaction networks: A mathematical framework to describe competitive growth under inclusion of metabolite cross-feeding
Source: PLoS Comput Biol. 2023 Aug 21;19(8):e1011402. doi: 10.1371/journal.pcbi.1011402 (PMC10470959; doi:10.1371/journal.pcbi.1011402)
Supplement: S1 Text — Appendix A: Logistic model for mono- and co-culture growth. Appendix B: Monod model for mono- and co-culture growth. Appendix C: Resource indifference. Appendix D: Vanishing waste concentration under permanent cross-feeding. Appendix E: Generalized consumer-resource model. Appendix F: RBN mass action kinetics. Appendix G: Inhibitory interactions. Appendix H: Metropolis-Hasting algorithm. (PDF) [file pcbi.1011402.s012.pdf]

Supplementary text to
Regulated bacterial interaction networks: A mathematical framework to describe competitive growth under inclusion of metabolite cross-feeding

Isaline Guex[1], Christian Mazza[1], Manupriyam Dubey[2] Renyi Li[2] Maxime Batsch[2] Jan Roelof van der Meer[2]

1 Dept. of Mathematics, University of Fribourg, Fribourg, Switzerland
2 Department of Fundamental Microbiology, University of Lausanne, Lausanne, Switzerland
For correspondence * christian.mazza@unifr.ch (CM)

Contents

Appendix A: Logistic model for mono- and co-culture growth
Appendix B: Monod model for mono- and co-culture growth
Appendix C: Resource indifference
Appendix D: Vanishing waste concentration under permanent cross-feeding
Appendix E: Generalized consumer-resource model
Appendix F: RBN mass action kinetics
Appendix G: Inhibitory interactions
Appendix H: Metropolis-Hasting algorithm
Supplementary references

Appendix A: Logistic model for mono- and co-culture growth

The logistic model assumes that growth can be described as a reaction involving a species S that enters in contact with a resource R, which it transforms to new cell biomass, and leading to cell division (duplication). This process is described by the reaction

$$S{+}R \xrightarrow{\kappa_s} 2S. \tag{1}$$

During cellular metabolism and biosynthesis, substances other than cell biomass are produced, which can leak outside the cell and which we (collectively) denote by W. Leaking substances may consist of regular metabolites in temporarily overflow (which the cell may take up again at a later stage), or specifically excreted compounds with a biological distinct function (e.g., signalling molecules or toxins), or wastes.

At this point, we assume that such product is a waste, which cannot be further used for growth by the same species.

$$S{+}R \xrightarrow{\kappa_w} S{+}W, \tag{2}$$

In these reactions, the kinetic rates $\kappa_s, \ \kappa_w \in \mathbb{R}^+$ refer to reaction rates; whereas $X = [S]$, $C = [R]$, $F = [W]$ are the mass concentrations of the species' biomass, of the resource and of the waste, respectively.

By using mass action law, the system of ordinary differential equations corresponding to these reactions for a monoculture is

$$\begin{aligned} \frac{dX(t)}{dt} &= \kappa_s X(t)C(t), \\ \frac{dC(t)}{dt} &= -(\kappa_s + \kappa_w)X(t)C(t), \\ \frac{dF(t)}{dt} &= \kappa_w X(t)C(t). \end{aligned} \tag{3}$$

At any time t, the sum of all components $\frac{dC(t)}{dt} + \frac{dF(t)}{dt} + \frac{dX(t)}{dt} = 0$, which leads to $C(t) + F(t) + X(t) = \text{constant}$. From $\frac{dF(t)}{dt} = \frac{\kappa_w}{\kappa_s}\frac{dX(t)}{dt}$ and $\frac{dC(t)}{dt} = -\frac{\kappa_s+\kappa_w}{\kappa_s}\frac{dX(t)}{dt}$, one deduces that $F(t) = \kappa_w \kappa_s X(t) + w_0 \frac{}{and}$C(t) = -$\kappa_s + \kappa_w \frac{}{\kappa_s X(t)+c_0}$, where w_0 and c_0 are constants to be determined. The initial states of the system are $F(0) = 0$, $C(0) = C_0 > 0$ and $X(0) = X^0 > 0$ therefore, $w_0 = -\frac{\kappa_w}{\kappa_s}X^0$ and $c_0 = C_0 + \frac{\kappa_s+\kappa_w}{\kappa_s}X^0$.

Because the total mass present in the system at any time t is constant and equals $C(t) + F(t) + X(t) = C_0 + X^0$, we can keep a single equation to describe the system. Hence, the development of the biomass of species S follows the logistic o.d.e.

$$\frac{dX(t)}{dt} = X(t)\kappa_s X_m \left(1 - \frac{X(t)}{\rho X_m}\right), \tag{4}$$

where $X_m = c_0 = C_0 + \frac{1}{\rho}X^0$ and $\rho = \frac{\kappa_s}{\kappa_s+\kappa_w}$.

We can approximate $X_m \approx C_0$ because the initial cell concentrations are negligibly low compared to the available resource concentration (in our experiments: 5 mM of succinate). We can now set the product of $\kappa_s X_m \approx \kappa_s C_0$ as μ_{max}, a constant that can be interpreted as the maximum growth rate of species S in the habitat defined by the experiment. ρ is a constant that represents the yield of substrate conversion into biomass for species S. The solution to (Eq. 4) is then given by the standard logistic growth curve

$$X(t) = \frac{\rho X_m X^0 e^{\mu_{max} t}}{\rho X_m - X^0 + X^0 e^{\mu_{max} t}}. \tag{5}$$

Logistic co-culture growth model without waste utilization

We can now expand the system to describe co-cultures with two different species using the same resource through the following CRN, where neither excreted waste products are consumed

$$\begin{aligned} S_1 + R &\xrightarrow{\kappa_{s_1}} 2S_1,\ S_1 + R \quad \xrightarrow{\kappa_{w_1}} S_1 + W_1, \\ S_2 + R &\xrightarrow{\kappa_{s_2}} 2S_2,\ S_2 + R \quad \xrightarrow{\kappa_{w_2}} S_2 + W_2, \end{aligned} \tag{6}$$

With the mass action law, this leads to the following set of o.d.e.s:

$$\begin{aligned} \frac{dX_1(t)}{dt} &= \kappa_{s_1} X_1(t) C(t), \\ \frac{dX_2(t)}{dt} &= \kappa_{s_2} X_2(t) C(t), \\ \frac{dW_1(t)}{dt} &= \kappa_{w_1} X_1(t) C(t), \\ \frac{dW_2(t)}{dt} &= \kappa_{w_2} X_2(t) C(t), \\ \frac{dC(t)}{dt} &= -(\kappa_{s_1} + \kappa_{w_1}) X_1(t) C(t) - ((\kappa_{s_2} + \kappa_{w_2})) X_2(t) C(t). \end{aligned} \tag{7}$$

We have $\frac{dW_i(t)}{dt} = \frac{\kappa_{w_i}}{\kappa_{s_i}} \frac{dX_i(t)}{dt}$ for $i = 1, 2$, therefore $W_i(t) = \frac{\kappa_{w_i}}{\kappa_{s_i}} S_i(t) + cst$. The yield for each species is defined by $\rho_i = \frac{\kappa_{s_i}}{\kappa_{s_i} + \kappa_{w_i}}$.

Then, if cell biomass concentrations of both species at start are negligibly low compared to the available resource, we can simplify by using the conservation relation $\frac{1}{\rho_1} X_1(t) + \frac{1}{\rho_2} X_2(t) + C(t) = \frac{1}{\rho_1} X_1^0 + \frac{1}{\rho_2} X_2^0 + C_0 \approx X_m$ from which we obtain

$$\begin{aligned} \frac{dX_1(t)}{dt} &= X_1(t) \kappa_{s_1} X_m \left(1 - \frac{\frac{1}{\rho_1} X_1(t) + \frac{1}{\rho_2} X_2(t)}{X_m}\right), \\ \frac{dX_2(t)}{dt} &= X_2(t) \kappa_{s_2} X_m \left(1 - \frac{\frac{1}{\rho_1} X_1(t) + \frac{1}{\rho_2} X_2(t)}{X_m}\right). \end{aligned} \tag{8}$$

By setting $\mu_{max}^{(i)} = \kappa_{s_i} X_m$ we obtain the system (Eq. 9) of a logistic model for two species with a single unique growth resource:

$$\begin{aligned} \frac{dX_1(t)}{dt} &= X_1(t) \mu_{max}^{(1)} \left(1 - \frac{\frac{1}{\rho_1} X_1(t) + \frac{1}{\rho_2} X_2(t)}{X_m}\right), \\ \frac{dX_2(t)}{dt} &= X_2(t) \mu_{max}^{(2)} \left(1 - \frac{\frac{1}{\rho_1} X_1(t) + \frac{1}{\rho_2} X_2(t)}{X_m}\right). \end{aligned} \tag{9}$$

The system of Eq. (9) has an explicit mathematical solution. First, notice that both solutions $X_i(t)$, $i = 1, 2$ are non-decreasing and bounded, and thus converge to positive equilibria $X_i^* > 0$ when $X_i^* > 0$. Next, observe that

$$\frac{dC(t)}{dt} \leq -(\kappa_{s_1} X_1^* + \kappa_{s_2} X_2^*) C(t),$$

in which case the Gronwall lemma can be used to define the upper bound

$$C(t) \leq C(0) \exp(-\beta t),$$

where $\beta = (\kappa_{s_1} X_1^* + \kappa_{s_2} X_2^*) > 0$. The concentration of substrate $C(t)$ decreases thus exponentially fast toward 0. The set of positive equilibria X_i^* is then given by the segment $I = \{(X_1^*, X_2^*);\ X_1^*/\rho_1 + X_2^*/\rho_2 = X_1(0)/\rho_1 + X_2(0)/\rho_2;\ X_i^* > 0,\ i = 1, 2\}$.

Finally, the maximum growth rate of species S_2 ($\mu_{max}^{(2)}$) and that of species S_1 ($\mu_{max}^{(1)}$) relate as follows:

$$\mu_{max}^{(2)} \frac{dX_1(t)}{X_1(t)dt} = \mu_{max}^{(1)} \frac{dX_2(t)}{X_2(t)dt},$$

thus

$$\mu_{max}^{(2)} \frac{d \ln X_1(t)}{dt} = \mu_{max}^{(1)} \frac{d \ln X_2(t)}{dt}. \tag{10}$$

Solving this last equation leads to

$$X_1(t) = \frac{X_1(0)}{X_2(0)^{\frac{\mu_{max}^{(1)}}{\mu_{max}^{(2)}}}} X_2(t)^{\frac{\mu_{max}^{(1)}}{\mu_{max}^{(2)}}}.$$

We can find the steady-state X_2^* as

$$x + \frac{\rho_2}{\rho_1} \frac{X_1(0)}{X_2(0) X_2(0)^{\frac{\mu_{max}^{(1)} - \mu_{max}^{(2)}}{\mu_{max}^{(2)}}}} x^{\frac{\mu_{max}^{(1)}}{\mu_{max}^{(2)}}} - \rho_2 X_m = 0. \tag{11}$$

Similarly, the steady-state of X_1^* equals

$$x + \frac{\rho_1}{\rho_2} \frac{X_2(0)}{X_1(0) X_1(0)^{\frac{\mu_{max}^{(2)} - \mu_{max}^{(1)}}{\mu_{max}^{(1)}}}} x^{\frac{\mu_{max}^{(2)}}{\mu_{max}^{(1)}}} - \rho_1 X_m = 0. \tag{12}$$

Therefore, the steady-states depend on the initial ratio between the two species, and also on their initial absolute abundances, since $X_1(0)^{\frac{\mu_{max}^{(2)} - \mu_{max}^{(1)}}{\mu_{max}^{(1)}}}$ and $X_2(0)^{\frac{\mu_{max}^{(1)} - \mu_{max}^{(2)}}{\mu_{max}^{(2)}}}$.

Appendix B: Monod model for mono and co-culture growth

In contrast to the logistic model, the Monod model assumes that a bacterial species S uses a unique resource R to form an intermediate complex P, which then gives rise both to a new cell and to waste products W. The main text demonstrates how this model is more accurate in describing co-culture growth of *P. veronii* and *P. putida*. In this case, equations (1) and (2) are rewritten as

$$\begin{aligned} S + R &\xrightarrow{\kappa_1} P \xrightarrow{\kappa_2} 2S, \\ P &\xrightarrow{\kappa_3} S + W. \end{aligned} \tag{13}$$

Let $X = [S] + [P],\ Y = [P],\ C = [R]$ and $F = [W]$ be the mass concentrations of the species S, the complex P, the resource R and the waste W, respectively. Let $X' = [S]$ denote the mass concentration of free S molecules that are not bound to R molecules in the intermediary complex P. Then the total bacterial biomass at time t

equals $X(t) = X'(t) + Y(t)$. From these reaction equations, we derive the following mass-action o.d.e.s

$$\begin{aligned}\frac{dX'(t)}{dt} &= -\kappa_1 X'(t)C(t) + (2\kappa_2 + \kappa_3)Y(t),\\ \frac{dY(t)}{dt} &= \kappa_1 X'(t)C(t) - (\kappa_2 + \kappa_3)Y(t),\\ \frac{dC(t)}{dt} &= -\kappa_1 X'(t)C(t)\\ \frac{dF(t)}{dt} &= \kappa_3 Y(t).\end{aligned} \tag{14}$$

To ensure consistency of units and denoting time in hours $[h]$, this would mean that κ_2 and κ_3 are given in $[h]^{-1}$, but that the unit of κ_1 is $([h][g]/[ml])^{-1}$.

Elimination of the intermediate complex P

Using a quasi-steady state approximation based on $\frac{1}{\kappa_1}\frac{dY(t)}{dt} = 0$, one obtains the simpler system

$$\begin{aligned} 0 &= (X(t) - Y(t))C(t) - \frac{(\kappa_2+\kappa_3)}{\kappa_1}Y(t),\\ \frac{dX(t)}{dt} &= \kappa_2 Y(t),\\ \frac{dC(t)}{dt} &= -\kappa_1(X(t) - Y(t))C(t),\\ \frac{dF(t)}{dt} &= \kappa_3 Y(t),\end{aligned} \tag{15}$$

which leads to a system with saturable consumption/degradation kinetics

$$\begin{aligned} Y(t) &= \frac{X(t)C(t)}{C(t) + \frac{\kappa_1+\kappa_2}{\kappa_1}},\\ \frac{dX(t)}{dt} &= \kappa_2 \frac{X(t)C(t)}{C(t) + \frac{\kappa_2+\kappa_3}{\kappa_1}}.\\ \frac{dC(t)}{dt} &= -(\kappa_2+\kappa_3)\frac{X(t)C(t)}{C(t) + \frac{\kappa_2+\kappa_3}{\kappa_1}},\\ \frac{dF(t)}{dt} &= \kappa_3 \frac{X(t)C(t)}{C(t) + \frac{\kappa_2+\kappa_3}{\kappa_1}}.\end{aligned} \tag{16}$$

$C(t) + F(t) + X(t) =$ constant and $\frac{dF(t)}{dt} = \frac{\kappa_3}{\kappa_2}\frac{dX(t)}{dt}$, $\frac{dC(t)}{dt} = -\frac{\kappa_2+\kappa_3}{\kappa_2}\frac{dX(t)}{dt}$, thus $F(t) = \frac{\kappa_3}{\kappa_2}X(t) + w_0$ and $C(t) = -\frac{\kappa_2+\kappa_3}{\kappa_2}X(t) + c_0$ where w_0, c_0 are constants to be determined.

By using the initial conditions $F(0) = 0$, $C(0) = C_0 > 0$ and $X(0) = X^0 > 0$, this leads to $w_0 = -\frac{\kappa_3}{\kappa_2}X^0$ and $c_0 = C_0 + \frac{\kappa_2+\kappa_3}{\kappa_2}X^0$. The total mass present in the system at any time t is constant and equals $C(t) + F(t) + X(t) = C_0 + X^0$. The development of the biomass of the species S then follows:

$$\frac{dX(t)}{dt} = X(t)\kappa_2 \frac{C_0 + \frac{1}{\rho}X(0) - \frac{1}{\rho}X(t)}{C_0 + \frac{1}{\rho}X(0) - \frac{1}{\rho}X(t) + \frac{\kappa_2+\kappa_3}{\kappa_1}}, \tag{17}$$

If we name $\rho = \frac{\kappa_2}{\kappa_2+\kappa_3}$, $X_m = c_0 = C_0 + \frac{1}{\rho}X^0$, $\mu_{max} = \kappa_2$ and $K_S = \frac{\kappa_2+\kappa_3}{\kappa_1}$ this leads to the Monod-model of substrate to biomass growth of Equation (18).

$$\frac{dX(t)}{dt} = \mu_{max}X(t)\frac{X_m - \frac{1}{\rho}X(t)}{X_m - \frac{1}{\rho}X(t) + K_S}, \tag{18}$$

where K_S is the resource concentration at which the growth rate of species S is half maximal.

Monod model for co-culture growth

In the case of two bacterial species (denoted by S_1 and S_2) and a single common resource (R) we obtain the CRNs (4) of the main text

$$\begin{aligned} &S_1 + R \xrightarrow{\kappa_{1_1}} P_1 \xrightarrow{\kappa_{1_2}} 2S_1,\ P_1 \xrightarrow{\kappa_{1_3}} S_1 + W_1, \\ &S_2 + R \xrightarrow{\kappa_{2_1}} P_2 \xrightarrow{\kappa_{2_2}} 2S_2,\ P_2 \xrightarrow{\kappa_{2_3}} S_2 + W_2. \end{aligned} \tag{19}$$

Proceeding the same way as for the system with one species and using the quasi-steady state approximation ($\frac{1}{\kappa_{i_1}}\frac{dY_i(t)}{dt} = 0$) we obtain the o.d.e.s:

$$\begin{aligned} &Y_1(t) = \frac{X_1(t)C(t)}{C(t) + \frac{\kappa_{1_2}+\kappa_{1_3}}{\kappa_{1_1}}},\ Y_2(t) = \frac{X_2(t)C(t)}{C(t) + \frac{\kappa_{2_2}+\kappa_{2_3}}{\kappa_{2_1}}}, \\ &\frac{dX_1(t)}{dt} = \kappa_{1_2}\frac{X_1(t)C(t)}{C(t) + \frac{\kappa_{1_2}+\kappa_{1_3}}{\kappa_{1_1}}},\ \frac{dX_2(t)}{dt} = \kappa_{2_2}\frac{X_2(t)C(t)}{C(t) + \frac{\kappa_{2_2}+\kappa_{2_3}}{\kappa_{2_1}}}, \\ &\frac{dC(t)}{dt} = -(\kappa_{1_2}+\kappa_{1_3})\frac{X_1(t)C(t)}{C(t) + \frac{\kappa_{1_2}+\kappa_{1_3}}{\kappa_{1_1}}} - (\kappa_{2_2}+\kappa_{2_3})\frac{X_2(t)C(t)}{C(t) + \frac{\kappa_{2_2}+\kappa_{2_3}}{\kappa_{2_1}}}, \\ &\frac{dF(t)}{dt} = \kappa_{1_3}\frac{X_1(t)C(t)}{C(t) + \frac{\kappa_{1_2}+\kappa_{1_3}}{\kappa_{1_1}}} + \kappa_{2_3}\frac{X_2(t)C(t)}{C(t) + \frac{\kappa_{2_2}+\kappa_{2_3}}{\kappa_{2_1}}}. \end{aligned} \tag{20}$$

where $X_i(t) = X_i'(t) + Y_i(t),\ X_i' = [S_i],\ Y_i = [P_i]$ and $C = [R]$. This kind of co-culture growth model with saturable degradation/consumption kinetics appears, e.g., in Ref. [1].

As before, we can make some simplifications because the variations of species, waste and resource concentrations are combinations of $Y_1(t)$ and $Y_2(t)$. Since $\frac{dC(t)}{dt} = -\frac{\kappa_{1_2}+\kappa_{1_3}}{\kappa_{1_2}}\frac{dX_1(t)}{dt} - \frac{\kappa_{2_2}+\kappa_{2_3}}{\kappa_{2_2}}\frac{dX_2(t)}{dt}$ this leads to $C(t) = -\frac{\kappa_{1_2}+\kappa_{1_3}}{\kappa_{1_2}}X_1(t) - \frac{\kappa_{2_2}+\kappa_{2_3}}{\kappa_{2_2}}X_2(t) + c_0$. Under the initial conditions $C(0) = C_0$ and $X_i(0) = X_i^0$, $c_0 = C_0 + \frac{\kappa_{1_2}+\kappa_{1_3}}{\kappa_{1_2}}X_1^0 + \frac{\kappa_{2_2}+\kappa_{2_3}}{\kappa_{2_2}}X_2^0$. By substituting $\mu_{max}^{(i)} = \kappa_{i_2}$, $\rho_1 = \frac{\kappa_{1_2}}{\kappa_{1_2}+\kappa_{1_3}}$ and $\rho_2 = \frac{\kappa_{2_2}}{\kappa_{2_2}+\kappa_{2_3}}$, we can simplify $c_0 = C_0 + \frac{1}{\rho_1}X_1^0 + \frac{1}{\rho_2}X_2^0$ that we call X_m, and further writing $K_S^{(i)} = \frac{\kappa_{i_2}+\kappa_{i_3}}{\kappa_{i_1}}$, we finally obtain

$$\begin{aligned} \frac{dX_1(t)}{dt} &= X_1(t)\mu_{max}^{(1)}\left(\frac{X_m - \frac{1}{\rho_1}X_1(t) - \frac{1}{\rho_2}X_2(t)}{X_m - \frac{1}{\rho_1}X_1(t) - \frac{1}{\rho_2}X_2(t) + K_S^{(1)}}\right), \\ \frac{dX_2(t)}{dt} &= X_2(t)\mu_{max}^{(2)}\left(\frac{X_m - \frac{1}{\rho_1}X_1(t) - \frac{1}{\rho_2}X_2(t)}{X_m - \frac{1}{\rho_1}X_1(t) - \frac{1}{\rho_2}X_2(t) + K_S^{(2)}}\right). \end{aligned} \tag{21}$$

In the special condition where $K_S^{(1)} = K_S^{(2)}$, we obtain again the relation (10) and then the steady-states for the species S_1 and S_2 can be found by solving equations (12) and (11), respectively.

Appendix C: Resource indifference

We can extend the two-species resource-competition based model for co-culture growth with exclusive substrates for each of the species R_1 and R_2. In this case, the CRN takes the form

$$\begin{aligned} S_1 + R_1 &\xrightarrow{\kappa_{1_1}} P_1 \xrightarrow{\kappa_{1_2}} 2S_1,\ P_1 \quad \xrightarrow{\kappa_{1_3}} S_1 + W_1, \\ S_2 + R_2 &\xrightarrow{\kappa_{2_1}} P_2 \xrightarrow{\kappa_{2_2}} 2S_2,\ P_2 \quad \xrightarrow{\kappa_{2_3}} S_2 + W_2. \end{aligned} \tag{22}$$

As in this case there is no competition for resource, the mass action kinetics would lead to biomass growth of species S_1 and S_2 with the same steady state values in co-culture as in monoculture (Appendix B). We show in the main text that such mathematical predictions are valid experimentally for co-cultures with dual exclusive substrates.

Appendix D: Vanishing waste concentration under permanent cross-feeding

We demonstrate here the mathematical unlikelihood that appearing metabolites during growth in co-cultures are reciprocally utilised in a linear concentration-dependent manner (see, e.g., Fig. 1C).

Let $I(t) = (F_1(t), F_2(t), C(t))$ be the line vector giving the concentrations of species W_1, W_2 and R at time $t > 0$. The mass action o.d.e. associated to the CRNs (2,3) of the main text, under permanent cross-feeding, $\phi \equiv 1$, is given by the sum of the o.d.e. associated to the pairwise Lotka-Volterra system and of the catalytic conversion reactions (Supplementary Figure S1). These conversion reactions can be described by a Laplace matrix L_{cat} where the entries are related to species W_1, W_2 and R, which are functions of the species S_1 and S_2 biomass concentrations X_1 and X_2:

$$L_{cat}(X_1, X_2) = \begin{bmatrix} -\tilde{\kappa}_{12} X_2 & \tilde{\kappa}_{12} X_2 & 0 \\ \tilde{\kappa}_{21} X_1 & -\tilde{\kappa}_{21} X_1 & 0 \\ \tilde{\kappa}_1 X_1 & \tilde{\kappa}_2 X_2 & -\tilde{\kappa}_1 X_1 - \tilde{\kappa}_2 X_2 \end{bmatrix}.$$

One can then define the matrix $Q = L_{cat} - D$, where D is the diagonal matrix

$$D(X_1, X_2) = \begin{bmatrix} \kappa_{12} X_2 & 0 & 0 \\ 0 & \kappa_{21} X_1 & 0 \\ 0 & & \kappa_1 X_1 + \kappa_2 X_2 \end{bmatrix},$$

which accounts for the consumption of species W_1, W_2 and R from S_1 and S_2. The mass action o.d.e. describing the time evolution of $I(t)$ is then given by

$$\frac{dI(t)}{dt} = I(t) Q(X_1(t), X_2(t)). \tag{23}$$

The development of species S_1 and S_2 biomass concentrations over time is then presented by

$$\begin{aligned} \frac{dX_1(t)}{dt} &= X_1(t)(\kappa_1 C(t) + \kappa_{21} F_2(t)), \\ \frac{dX_2(t)}{dt} &= X_2(t)(\kappa_2 C(t) + \kappa_{12} F_1(t)). \end{aligned} \tag{24}$$

The mass action o.d.e. associated with the CRNs (2,3) of the main text under permanent cross-feeding, is given by the system of o.d.e. (23,24). This system is

conservative so that the biomass $X_i(t)$, $i = 1, 2$ remains bounded. The derivative of these functions being non-negative, it follows that both biomass concentrations converge toward positive limiting values $X_i(\infty) > 0$.

Proposition 1. The solutions $X_i(t)$ with $X_i(0) > 0$, $i = 1, 2$ of the mass action o.d.e. (23,24) are non-decreasing and bounded, and converge toward positive limits $X_i(\infty) > 0$. There exists a positive constant $\lambda > 0$ such that $C(t) \leq C(0)\exp(-\lambda t)$. Consider the linear o.d.e. (23), where the concentration functions $X_1(t)$, $X_2(t)$, $F_1(t)$, $F_2(t)$ and $C(t)$ solve the mass-action o.d.e. (23,24). Then the origin $I = 0$ is globally asymptotically stable for the positive orthant. The manifold of states $(X_1, X_2, F_1 = 0, F_2 = 0, C = 0)$ with $X_1 + X_2 = C(0) + X_1(0) + X_2(0)$ is an equilibrium manifold.

Proposition 1, which is proven below, shows that, starting from any initial condition for the various concentrations with positive $X_i(0) > 0$, the waste concentrations $F_i(t)$ and $C(t)$ will ultimately converge toward 0, so that the resulting consumer steady-state concentrations $X_i(t)$ will converge toward some X_i^* with $X_1^* + X_2^* = X_1(0) + X_2(0) + C(0)$. Hence, the cross-feeding mechanism of the generalized consumer-resource model (see Refs. [2, 3])) leads to vanishing waste steady-state concentrations.

The linear equation obtained by setting $dX_i/dt = 0$ further implies that the critical points with $X_i > 0$ are such that $C = F_1 = F_2 = 0$. This small argument might lead to a misunderstanding about the true content of Proposition 1. The convergence of all orbits towards equilibrium like $(X_1^*, X_2^*, 0, 0, 0)$ follows from the structure of the matrix $Q = L_{cat} - D$: L_{cat} is a Laplace matrix which preserves mass, that is, describes how metabolite molecules are transformed into another metabolite, while D describes consumption of metabolite molecules by consumers, and thus induces total metabolite mass decrease. We consider a more general model by adding arbitrary nonlinear terms $f_i(Z(t))$, $i = 1, 2$, to (24), where $Z(t) = (X_1(t), X_2(t), C(t), F_1(t), F_2(t))$. For example, we might also consider competition between consumers, and these new functions might take the form

$$f_i(Z(t)) = -\sum_{j \neq i} C_{ij}^{comp} X_i(t) X_j(t) + r_i X_i(t),$$

for competition coefficients $C_{ij}^{comp} > 0$ and intrinsic growth rates r_i. Another possible model might include predation between consumers. The new version of (24) is

$$\begin{aligned} \frac{dX_1(t)}{dt} &= X_1(t)(\kappa_1 C(t) + \kappa_{21} F_2(t)) + f_1(Z(t)), \\ \frac{dX_2(t)}{dt} &= X_2(t)(\kappa_2 C(t) + \kappa_{12} F_1(t)) + f_1(Z(t)). \end{aligned} \tag{25}$$

Proposition 2. Assume that the o.d.e. (23 and 25) admits unique solutions such that X_1 and X_2 converge toward positive limiting values. Let $I(t) = (F_1(t), F_2(t), C(t))$ and consider the linear o.d.e. (23), where the concentration function $Z(t)$ solves the mass-action o.d.e. (23,25). Then the origin $I = 0$ is globally asymptotically stable for the positive orthant.

Proof of Proposition 1 and 2:

We begin with the proof of Proposition 1: A simple analysis of the mass-action o.d.e. associated with the CRN reveals that the equilibria (X_1, X_2, C, F_1, F_2) are such that $C = 0$. We thus study the solutions of (24).One can check that manifolds of equilibria exist where either $X_1 = 0$ or $X_2 = 0$, but our equations lead that $dX_i(t)/dt \geq 0$, so that

imposing initial conditions with $X_i(0) > 0$, $i = 1, 2$, the orbits of the o.d.e. will never reach theses invariant manifolds and, using the fact that they are bounded and non-decreasing (see, e.g. [4]), converge toward positive equilibria $X_i(\infty) > 0$.

We then show that $C(t)$ converges exponentially fast toward 0 as $t \to \infty$. We have seen that $\lim_{t\to\infty} X_i(t) = X_i(\infty) > X_i(0)$, and that $X_i(t)$ is non-decreasing, $i = 1, 2$, so that

$$\begin{aligned}\frac{dC(t)}{dt} &= -((\kappa_1 + \tilde{\kappa}_1)X_1(t) + (\kappa_2 + \tilde{\kappa}_2)X_2(t))C(t)\\ &\leq -((\kappa_1 + \tilde{\kappa}_1)X_1(0) + (\kappa_2 + \tilde{\kappa}_2)X_2(0))C(t) = -\lambda C(t),\end{aligned}$$

with $C(t) > 0$, $\forall t \geq 0$, where $\lambda = (\kappa_1 + \tilde{\kappa_1})X_1(0) + (\kappa_2 + \tilde{\kappa}_2)X_2(0) > 0$. Gronwall's inequality then yields the upper bound

$$C(t) \leq C(0)\exp(-\lambda t),\ t \geq 0.$$

We use these estimates to prove that the origin is globally asymptotically stable for the o.d.e. (23) on the positive orthant.

The following arguments finish the proof of Proposition 1 and prove Proposition 2. The CRN given in (2,3) of the main text contains a subsystem involving catalytic conversion, see Fig. 1 B, main text, whose dynamics is well described by a Laplace matrix L_{cat} where the entries are related to species W_1, W_2 and R, which depends on the S_1 and S_2 concentrations X_1 and X_2

$$L_{cat}(X_1, X_2) = \begin{bmatrix} -\tilde{\kappa}_{12}X_2 & \tilde{\kappa}_{12}X_2 & 0 \\ \tilde{\kappa}_{21}X_1 & -\tilde{\kappa}_{21}X_1 & 0 \\ \tilde{\kappa}_1 X_1 & \tilde{\kappa}_2 X_2 & -\tilde{\kappa}_1 X_1 - \tilde{\kappa}_2 X_2 \end{bmatrix}.$$

As seen previously, both $X_i(t)$ converge toward positive limits $X_i(\infty)$, so that one can consider the limiting Laplace matrix

$$L_{cat,\infty} = \begin{bmatrix} -\tilde{\kappa}_{12}X_2(\infty) & \tilde{\kappa}_{12}X_2(\infty) & 0 \\ \tilde{\kappa}_{21}X_1(\infty) & -\tilde{\kappa}_{21}X_1(\infty) & 0 \\ \tilde{\kappa}_1 X_1(\infty) & \tilde{\kappa}_2 X_2(\infty) & -\tilde{\kappa}_1 X_1 - \tilde{\kappa}_2 X_2(\infty) \end{bmatrix},$$

and, similarly, one arrives at the limiting diagonal matrix

$$D_\infty = \begin{bmatrix} \kappa_{12}X_2(\infty) & 0 & 0 \\ 0 & \kappa_{21}X_1(\infty) & 0 \\ 0 & & \kappa_1 X_1(\infty) + \kappa_2 X_2(\infty) \end{bmatrix},$$

Let $I(t) = (I_1(t), I_2(t), I_3(t)) = (F_1(t), F_2(t), C(t))$ be the line vector giving their concentrations at time $t > 0$.The mass action kinetics leads to

$$\frac{dI(t)}{dt} = I(t)Q, \tag{26}$$

where $Q = L_{cat}(X_1(t), X_2(t)) - D$.

Using the convergence of $X_i(t)$, this last o.d.e. is close to the linear autonomous o.d.e.

$$\frac{dI(t)}{dt} = I(t)Q_\infty, \tag{27}$$

where $Q_\infty = L_{cat,\infty} - D_\infty$.

The structure of the o.d.e. (26) suggests that its asymptotic behaviour is close to that of the autonomous one. First notice that

$$\frac{dI(t)}{dt} = I(t)Q_\infty + g(t, I(t)),$$

where

$$g(t,y) = y(Q(t) - Q_\infty).$$

The entries of the matrix $Q(t) - Q_\infty$ are products involving the various kinetic constants or sums of them, that are bounded by some positive constant $K^0 > 0$, so that

$$||g(t,y)|| \leq 3K^0 \sup_k |y_k| \max\{|X_1(t) - X_1(\infty)|, |X_2(t) - X_2(\infty)|\}.$$

It follows that, using the convergence of the X_i and the boundedness of all functions, we arrive at $||g(t,y)|| \leq \gamma(t)$, for all y in a compact B that depend on the initial concentrations, with $\gamma(t) \to 0$ as $t \to \infty$. Then Corolloray 3.3. of [5] shows that all solutions of (26) converge toward 0 when the origin is globally asymptotically stable for the autonomous o.d.e., as required for Proposition 1.

To show that the origin is globally asymptotically stable for the o.d.e. (27), is it sufficient to prove that all the real parts of the eigenvalues of the matrix Q_∞ are negative. This is seen using the Levy-Hadamard Theorem, which states that all of the eigenvalues are contained in the union of the discs of center $(Q_\infty)_{kk}$ of radius $\sum_{m\neq k}(Q_\infty)_{km}$. A direct computation shows that this holds true.

Appendix E: Generalized consumer-resource model

We present here the generalized consumer-resource model described in [2] and [6]. Let W_k, $k = 1, \dots, M$ be the byproducts or waste species and let S_i, $i = 1, \dots, n$, be the set of consumers. Let c_{ik} and l_k be the rate at which species S_i uptakes resource W_k and the leakage factor for resource k with $0 \leq l_k \leq 1$. Let σ be a single-valued function encoding the relationship between resource supply levels and uptake rates. Typically, σ is linear or a sigmoidal function of Hill type. Let m_i be the minimal energy required for growth of species S_i. The biomass of species S_i at time t is denoted by $N_i(t)$, and the abundance of waste W_k is denoted by $R_k(t)$. The o.d.e. describing the time evolution of N_i is

$$\frac{dN_i}{dt} = g_i N_i \Big(\sum_k (1 - l_k) q_k \sigma(c_{ik} R_k) - m_i \Big), \tag{28}$$

where the function $q = (q_k)$ might be, for each resource W_k, the maximal ATP yield of resource W_k, and g_i is a positive proportionality factor. Concerning the time evolution of resources, let, for each species S_i, the stoichiometric matrix $D = (D_{kl})_{1\leq k,l\leq M}$ encode the number of molecules of resource k secreted to the environment per molecule of resource l taken up. The secretion matrix $P = D^T$ is assumed to be stochastic. Within this modelling framework, the production rate of resource W_k from secretions is

$$\Lambda_k = \sum_{l,i} l_k p_{lk} \frac{q_l}{q_k} \sigma(c_{il} R_l) N_i.$$

The o.d.e. governing the time evolution of resources is then

$$\frac{dR_k}{dt} = \frac{K_k - R_k}{\tau_k} - \sum_i \sigma(c_{ik} R_k) N_i + \Lambda_k, \tag{29}$$

where K_k is the initial abundance of resource W_k, and τ_k is the transfer rate during batch culture passaging. We assume here that $\tau_k >> 1$, so that the first term $(K_k - R_k)/\tau_k$ can be neglected.

We show that the mass-action o.d.e. associated to the CRN (2,3) of the main text with permanent cross-feeding $\phi \equiv 1$

$$\begin{aligned} S_1 + R &\xrightarrow{\kappa_1} 2S_1, \; S_1 + R \quad \xrightarrow{\tilde{\kappa}_1} S_1 + W_1, \\ S_2 + R &\xrightarrow{\kappa_2} 2S_2, \; S_2 + R \quad \xrightarrow{\tilde{\kappa}_2} S_2 + W_2, \end{aligned} \tag{30}$$

$$\begin{aligned} S_1 + W_2 &\xrightarrow{\kappa_{21}} 2S_1, \quad S_1 + W_2 \quad \xrightarrow{\tilde{\kappa}_{21}} S_1 + W_1, \\ S_2 + W_1 &\xrightarrow{\kappa_{12}} 2S_2, \quad S_2 + W_1 \quad \xrightarrow{\tilde{\kappa}_{12}} S_2 + W_2. \end{aligned} \tag{31}$$

can be recast as a generalized consumer-resource model, but involves fewer parameters. The mass action kinetics o.d.e. associated with eq.(30, 31) is given by

$$\begin{aligned} \frac{dX_1(t)}{dt} &= \kappa_1 X_1(t)F_3(t) + \kappa_{21}X_1(t)F_2(t), \\ \frac{dX_2(t)}{dt} &= \kappa_2 X_2(t)F_3(t) + \kappa_{12}X_2(t)F_1(t), \\ \frac{dF_1(t)}{dt} &= \tilde{\kappa}_1 X_1(t)F_3(t) - (\kappa_{12} + \tilde{\kappa}_{12})X_2(t)F_1(t) + \tilde{\kappa}_{21}X_1(t)F_2(t), \\ \frac{dF_2(t)}{dt} &= \tilde{\kappa}_2 X_2(t)F_3(t) - (\kappa_{21} + \tilde{\kappa}_{21})X_1(t)F_2(t) + \tilde{\kappa}_{12}X_2(t)F_1(t), \\ \frac{dF_3(t)}{dt} &= -(\kappa_1 + \tilde{\kappa}_1)X_1(t)F_3(t) - (\kappa_2 + \tilde{\kappa_2})X_2(t)F_3(t), \end{aligned} \tag{32}$$

where X_i are the concentrations of species S_i, $i = 1, 2$, F_k, $k = 1, 2$ are the concentrations of waste W_k, $k = 1, 2$, and F_3 the concentration of the resource R.

This o.d.e. is similar to that defined in (28,29). Appendix D considers these two systems of differential equations and arrives at the non-identifiability of generalized consumer-resource models within our framework. One can find a one dimensional manifold of parameters g_i, and l_k, q_k, p_{lk} and c_{ik} so that (28,29) and (44) coincide for given parameters κ_i, $\tilde{\kappa}_i$, κ_{ki} and $\tilde{\kappa}_{ki}$.

The network structure suggests that the secretion matrix P is of the form

$$P = \begin{bmatrix} 0 & p_{12} & 0 \\ p_{21} & 0 & 0 \\ p_{31} & p_{32} & 0 \end{bmatrix}$$

The assumed stochasticity of the secretion matrix imposes that $p_{12} = p_{21} = 1$ and $p_{31} + p_{32} = 1$, and the resulting generalized consumer-resource model becomes

$$\begin{aligned} \frac{dN_1}{dt} &= g_1 N_1((1 - l_3)q_5 c_{13} R_3 + (1 - l_2)q_2 c_{12} R_2), \\ \frac{dN_2}{dt} &= g_2 N_2((1 - l_3)q_3 c_{23} R_3 + (1 - l_1)q_1 c_{21} R_1), \\ \frac{dR_1}{dt} &= -c_{21} R_1 N_2 + \Lambda_1, \\ \frac{dR_2}{dt} &= -c_{12} R_2 N_1 + \Lambda_2, \\ \frac{dR_3}{dt} &= -c_{13} R_3 N_1 - c_{23} R_3 N_2, \end{aligned} \tag{33}$$

where

$$\begin{aligned} \Lambda_1 &= l_2 \frac{q_2}{q_1} c_{12} R_2 N_1 + l_3 p_{31} \frac{q_3}{q_1} c_{13} R_3 N_1, \\ \Lambda_2 &= l_1 \frac{q_1}{q_2} c_{21} R_1 N_2 + l_3 p_{32} \frac{q_3}{q_2} c_{23} R_3 N_2, \end{aligned}$$

and $\Lambda_3 = 0$. One then compares the two o.d.e.s to arrive at the two sets of equations which correspond to Fig 1 A and B of the main text:

$$\kappa_1 = g_1(1 - l_3)q_3 c_{13}, \quad \kappa_{21} = g_1(1 - l_2)q_2 c_{12},$$

$$\kappa_2 = g_2(1-l_3)q_3c_{23}, \quad \kappa_{12} = g_2(1-l_1)q_1c_{21},$$

and

$$\tilde{\kappa}_1 = l_3p_{31}\frac{q_3}{q_1}c_{13}, \quad \tilde{\kappa}_{21} = l_2\frac{q_2}{q_1}c_{12},$$

$$\tilde{\kappa}_2 = l_2p_{32}\frac{q_3}{q_2}c_{23}, \quad \tilde{\kappa}_{12} = l_1\frac{q_1}{q_2}c_{21}.$$

We suppose are being given the parameters of (32). Then we describe the set of parameters g_1, g_2, l_k, q_k and c_{ik} that solve the above set of equations. We first observe that necessarily

$$c_{13} = \kappa_1 + \tilde{\kappa}_1, \quad c_{23} = \kappa_2 + \tilde{\kappa}_2,$$

$$c_{21} = \kappa_{12} + \tilde{\kappa}_{12}, \quad c_{12} = \kappa_{21} + \tilde{\kappa}_{21}.$$

We then focus on the second set of equations using notions from algebraic potential theory associated to the directed graph given in Fig. S1 of node set $V = \{R_1,\ R_2,\ R_3\}$ and edge set $E = \{f_1,\ f_2,\ f_3,\ f_4\}$. Set for convenience

$$y_1^{-1} = \frac{\tilde{\kappa}_1}{l_3p_{31}c_{13}}, \quad y_2^{-1} = \frac{\tilde{\kappa}_{21}}{l_2c_{12}}, \quad y_3^{-1} = \frac{\tilde{\kappa}_2}{l_3p_{32}c_{23}}, \quad y_4^{-1} = \frac{\tilde{\kappa}_{12}}{l_1c_{21}}.$$

Let $L_y = (\ln(y_1), \ln(y_2), \ln(y_3), \ln(y_4))^T$, and consider the incidence matrix

$$I = \begin{bmatrix} 1 & 1 & 0 & -1 \\ 0 & -1 & 1 & 1 \\ -1 & 0 & -1 & 0 \end{bmatrix}$$

Then the second block of equation becomes

$$L_y = I^T L_q, \tag{34}$$

where $L_q = (\ln(q_1), \ln(q_2), \ln(q_3))^T$, which corresponds to a Dirichlet problem, see [7]. The last equation has a solution when $L_y \in \mathrm{Im}(I^T)$, which is the orthogonal complement to the cycle space of the graph (Proposition 4.1 of [7]). The two cycles of the graph are (f_4, f_2) and $(f_1, f_4, -f_3)$ of characteristic vectors $(0,1,0,1)$ and $(1,0,-1,1)$. The orthogonality relation yields the two conditions

$$\frac{\tilde{\kappa}_{21}}{\tilde{\kappa}_{21}+\kappa_{21}}\frac{\tilde{\kappa}_{12}}{\tilde{\kappa}_{12}+\kappa_{12}} = l_1l_2, \tag{35}$$

and

$$\frac{\tilde{\kappa}_1}{\tilde{\kappa}_1+\kappa_1}\frac{\tilde{\kappa}_2+\kappa_2}{\tilde{\kappa}_2}\frac{\tilde{\kappa}_{12}}{\tilde{\kappa}_{12}+\kappa_{12}} = \frac{p_{31}}{p_{32}}l_1. \tag{36}$$

From here, one can solve explicitly (34) using the general theory as in [7], Section 9, or work directly to arrive at the solutions when both (35) and (36) are satisfied.

The parameters l_1, l_2, q_1 and q_3 can be deduced from the other. Set for convenience

$$q_{21} = \frac{\tilde{\kappa}_{21}}{\tilde{\kappa}_{21}+\kappa_{21}}, \quad q_{12} = \frac{\tilde{\kappa}_{12}}{\tilde{\kappa}_{12}+\kappa_{12}},$$

and

$$q_{31} = \frac{\tilde{\kappa}_1}{\tilde{\kappa}_1+\kappa_1}, \quad q_{32} = \frac{\tilde{\kappa}_2}{\tilde{\kappa}_2+\kappa_2}.$$

Hence, with these notations,

$$l_1 = \frac{q_{31}q_{12}}{q_{32}}\frac{p_{32}}{p_{31}}, \tag{37}$$

and

$$l_2 = \frac{q_{21} q_{32}}{q_{31}} \frac{p_{31}}{p_{32}}, \tag{38}$$

$$q_1 = \frac{q_2 l_2}{q_{21}}, \tag{39}$$

and

$$q_3 = \frac{q_{32}}{p_{32} l_3} q_2. \tag{40}$$

One can then focus on the first set of equations, which becomes after some computations,

$$\frac{\kappa_1}{c_{13} q_{32}} = g_1 \frac{1 - l_3}{l_3} \frac{q_2}{p_{32}},$$
$$\frac{\kappa_{21}}{c_{12}} = g_1 (1 - \frac{q_{21} q_{32}}{q_{31}} \frac{p_{31}}{1 - p_{31}}) q_2,$$
$$\frac{\kappa_2}{c_{23} q_{32}} = g_2 \frac{1 - l_3}{l_3} \frac{q_2}{p_{32}},$$
$$\frac{\kappa_{12} q_{21}}{c_{21}} = g_2 (\frac{q_{21} q_{32}}{q_{31}} \frac{p_{31}}{p_{32}} - q_{21} q_{12}) q_2.$$

Let A, B, C and D be the following functions of p_{31} (recall that $p_{31} + p_{32} = 1$)

$$A = \frac{\kappa_1}{c_{13} q_{32}} (1 - p_{31}), \quad B = \frac{\kappa_{21}}{c_{12}} \frac{1}{1 - \frac{q_{21} q_{32}}{q_{31}} \frac{p_{31}}{1 - p_{31}}},$$

$$C = \frac{\kappa_2}{c_{23} q_{32}} (1 - p_{31}), \quad D = \frac{\kappa_{12} q_{21}}{c_{21}} \frac{1}{(\frac{q_{21} q_{32}}{q_{31}} \frac{p_{31}}{p_{32}} - q_{21} q_{12})}.$$

The equation is then equivalent to

$$A = g_1 (\frac{1}{l_3} - 1) q_2, \quad B = g_1 q_2, \quad C = g_2 (\frac{1}{l_3} - 1) q_2, \quad D = g_2 q_2$$

Considering the ratios A/B and C/D, one deduces the relations

$$l_3 = \frac{1}{1 + \frac{A}{B}} = \frac{1}{1 + \frac{C}{D}}. \tag{41}$$

One thus must check that there is some p_{31} satisfying (41) When $A \neq -B$ and $C \neq -D$, this is equivalent to $\frac{A}{B} = \frac{C}{D}$. which corresponds to :

$$\frac{\kappa_1}{\kappa_1 + \tilde{\kappa}_1} \frac{\kappa_2 + \tilde{\kappa}_2}{\tilde{\kappa}_2} \frac{\kappa_{21} + \tilde{\kappa}_{21}}{\kappa_{21}} (1 - p_{31}) - \frac{\tilde{\kappa}_{21}}{\kappa_{21}} = \frac{\kappa_2^2}{\tilde{\kappa}_2 (\tilde{\kappa}_2 + \kappa_2)} \frac{\kappa_{12} + \tilde{\kappa}_{12}}{\kappa_{12}} \frac{\tilde{\kappa}_1 + \kappa_1}{\tilde{\kappa}_1} p_{31} - \frac{\kappa_2 \tilde{\kappa} 12}{\tilde{\kappa}_2 \kappa_{12}} (1 - p_{31}),$$

wich can be solved to arrive at

$$p_{31} = \frac{\frac{\kappa_1}{\kappa_1 + \tilde{\kappa}_1} \frac{\kappa_2 + \tilde{\kappa}_2}{\tilde{\kappa}_2} \frac{\kappa_{21} + \tilde{\kappa}_{21}}{\kappa_{21}} + \frac{\kappa_2 \tilde{\kappa}_{12}}{\tilde{\kappa}_2 \kappa_{12}}}{\frac{\kappa_1}{\kappa_1 + \tilde{\kappa}_1} \frac{\kappa_2 + \tilde{\kappa}_2}{\tilde{\kappa}_2} \frac{\kappa_{21} + \tilde{\kappa}_{21}}{\kappa_{21}} + \frac{\tilde{\kappa}_{21}}{\kappa_{21}} + \frac{\kappa_2^2}{\tilde{\kappa}_2 (\tilde{\kappa}_2 + \kappa_2)} \frac{\kappa_{12} + \tilde{\kappa}_{12}}{\kappa_{12}} \frac{\tilde{\kappa}_1 + \kappa_1}{\tilde{\kappa}_1} + \frac{\kappa_2 \tilde{\kappa}_{12}}{\tilde{\kappa}_2 \kappa_{12}}},$$

Hence,

$$g_1 = \frac{A}{q_2 (\frac{1}{l_3} - 1)}, \quad g_2 = \frac{D}{q_2}.$$

One sees that 1) the constants g_i, $i = 1, 2$ are necessary and that 2) the model is not identifiable since this last system has a one dimensional solution set.

Appendix F: RBN mass action kinetics

We consider the following simplified CRN given in (2,3) of the main text

$$\begin{aligned} S_1 + R &\xrightarrow{\kappa_1} 2S_1, \;\; S_1 + R \quad \xrightarrow{\tilde{\kappa}_1} S_1 + W_1, \\ S_2 + R &\xrightarrow{\kappa_2} 2S_2, \;\; S_2 + R \quad \xrightarrow{\tilde{\kappa}_2} S_2 + W_2, \end{aligned} \tag{42}$$

$$\begin{aligned} S_1 + W_2 &\xrightarrow{\phi(\mathrm{F_2 - T_{W_2}})\kappa_{21}} 2S_1, \quad S_1 + W_2 \quad \xrightarrow{\phi(\mathrm{F_2 - T_{W_2}})\tilde{\kappa}_{21}} S_1 + W_1, \\ S_2 + W_1 &\xrightarrow{\phi(\mathrm{F_1 - T_{W_1}})\kappa_{12}} 2S_2, \quad S_2 + W_1 \quad \xrightarrow{\phi(\mathrm{F_1 - T_{W_1}})\tilde{\kappa}_{12}} S_2 + W_2, \end{aligned} \tag{43}$$

of associated mass-action o.d.e.,

$$\begin{aligned} \frac{dX_1(t)}{dt} &= \kappa_1 X_1(t)C(t) + \phi(F_2 - T_{W_2})\kappa_{21} X_1(t)F_2(t), \\ \frac{dX_2(t)}{dt} &= \kappa_2 X_2(t)C(t) + \phi(F_1 - T_{W_1})\kappa_{12} X_2(t)F_1(t), \\ \frac{dF_1(t)}{dt} &= \tilde{\kappa}_1 X_1(t)C(t) - \phi(F_1 - T_{W_1})(\kappa_{12} + \tilde{\kappa}_{12})X_2(t)F_1(t) + \phi(F_2 - T_{W_2})\tilde{\kappa}_{21} X_1(t)F_2(t), \\ \frac{dF_2(t)}{dt} &= \tilde{\kappa}_2 X_2(t)C(t) - \phi(F_2 - T_{W_2})(\kappa_{21} + \tilde{\kappa}_{21})X_1(t)F_2(t) + \phi(F_1 - T_{W_1})\tilde{\kappa}_{12} X_2(t)F_1(t), \\ \frac{dC(t)}{dt} &= -(\kappa_1 + \tilde{\kappa}_1)X_1(t)C(t) - (\kappa_2 + \tilde{\kappa_2})X_2(t)C(t). \end{aligned} \tag{44}$$

Assume e.g. that the activation function of the RBN is $\phi_\varepsilon(x) = x/(2\varepsilon) + 1/2$ for $|x| \le \varepsilon$, $\phi_\varepsilon(x) = 0$ for $x \le -\varepsilon$ and $\phi_\varepsilon(x) = 1$ when $x \ge \varepsilon$. We look for critical points $(X_1^*, X_2^*, F_1^*, F_2^*, C^*)$ of the o.d.e. (44) with $X_i^* > 0$, $i = 1, 2$. Such critical point must satisfy

$$\kappa_1 X_1^* C^* + \phi(F_2^* - T_{W_2})\kappa_{21} X_1^* F_2^* = 0.$$

The solution of mass action being non-negative when starting from non-negative initial conditions (see [4]), we get that necessarily $C^* = 0$ and that either $F_2^* = 0$ or $\phi_\varepsilon(F_2^* - T_{W_2}) = 0$, that is $F_2^* \le T_{W_2} - \varepsilon$. Proceeding similarly for the remaining equations, one arrives at the following manifold I of equilibria

$$I = \{(X_1^*, X_2^*, F_1^*, F_2^*, C^*);\; X_i^* > 0,\; F_i^* \le T_i - \varepsilon,\; i = 1,\; 2,\; C^* = 0\}.$$

RBN mass action kinetics with intermediate complexes

We consider the same system as in Appendix E but including the creation of the intermediate complex P for each species.

$$\begin{aligned} S_1 + R &\xrightarrow{\kappa_{1_1}} P_1 \xrightarrow{\kappa_{1_2}} 2S_1, \quad P_1 \quad \xrightarrow{\kappa_{1_3}} S_1 + W_1, \\ S_2 + R &\xrightarrow{\kappa_{2_1}} P_2 \xrightarrow{\kappa_{2_2}} 2S_2, \quad P_2 \quad \xrightarrow{\kappa_{2_3}} S_2 + W_2, \\ S_1 + W_2 &\xrightarrow{\phi(\mathrm{F_2 - T_{W_2}})\kappa_{1_1}} P_1, \\ S_2 + W_1 &\xrightarrow{\phi(\mathrm{F_1 - T_{W_1}})\,\kappa_{2_1}} P_2. \end{aligned} \tag{45}$$

Applying the mass action law to the system (45), we obtain the o.d.e.,

$$
\begin{aligned}
\frac{dS_1(t)}{dt} &= (2\kappa_{1_2} + \kappa_{1_3})P_1(t) - \kappa_{1_1}S_1(t)R(t) - \phi(F_2 - T_{W_2})\kappa_{1_1}S_(t)1W_2(t),\\
\frac{dS_2(t)}{dt} &= (2\kappa_{2_2} + \kappa_{2_3})P_2(t) - \kappa_{2_1}S_2(t)R(t) - \phi(F_1 - T_{W_1})\kappa_{2_1}S_2(t)W_1(t),\\
\frac{dP_1(t)}{dt} &= -(\kappa_{1_2} + \kappa_{1_3})P_1(t) + \kappa_{1_1}S_1(t)R(t) + \phi(F_2 - T_{W_2})\kappa_{1_1}S_1(t)W_2(t),\\
\frac{dp_2(t)}{dt} &= -(\kappa_{2_2} + \kappa_{2_3})P_2(t) + \kappa_{2_1}S_2(t)R(t) + \phi(F_1 - T_{W_1})\kappa_{2_1}S_2(t)W_1(t),\\
\frac{dW_1(t)}{dt} &= \kappa_{1_3}P_1(t) - \phi(F_1 - T_{W_1})\kappa_{2_1}S_2(t)W_1(t),\\
\frac{dW_2(t)}{dt} &= \kappa_{2_3}P_2(t) - \phi(F_2 - T_{W_2})\kappa_{1_1}S_1(t)W_2(t),\\
\frac{dR(t)}{dt} &= -\kappa_{1_1}S_1(t)R(t) - \kappa_{2_1}S_2(t)R(t).
\end{aligned}
\tag{46}
$$

Appendix G: Inhibitory interactions

In contrast to the models simulating metabolite cross-feeding, we simulate a system where species produce metabolites causing potential reciprocal inhibition of growth. We developed two scenarios. In the first, we assume that each species can produce a byproduct that inhibits the other (reciprocal inhibition). In the second, we assume unilateral inhibition (only one of the species inhibits the other).

As for the models with metabolite cross-feeding, we describe inhibition using a Hill function, which is dependent on byproduct concentrations. The general description of the inhibitory CRN is:

$$
\begin{aligned}
S_1 + R &\xrightarrow{\kappa_{1_1}} P_1 \xrightarrow{\kappa_{1_2}} 2S_1, \quad P_1 \xrightarrow{\kappa_{1_3}} S_1 + W_1,\\
S_2 + R &\xrightarrow{\kappa_{2_1}} P_2 \xrightarrow{\kappa_{2_2}} 2S_2, \quad P_2 \xrightarrow{\kappa_{2_3}} S_2 + W_2,\\
S_1 + W_2 &\xrightarrow{\phi(\mathrm{F_2} - \mathrm{T_{W_2}})\kappa_{1_1}} \emptyset,\\
S_2 + W_1 &\xrightarrow{\phi(\mathrm{F_1} - \mathrm{T_{W_1}})\,\kappa_{2_1}} \emptyset.
\end{aligned}
\tag{47}
$$

Applying the mass action law to the system (47), we obtain the o.d.e.,

$$
\begin{aligned}
\frac{dS_1(t)}{dt} &= (2\kappa_{1_2} + \kappa_{1_3})P_1(t) - \kappa_{1_1}S_1(t)R(t) - \phi(F_2 - T_{W_2})\kappa_{1_1}S_(t)1W_2(t),\\
\frac{dS_2(t)}{dt} &= (2\kappa_{2_2} + \kappa_{2_3})P_2(t) - \kappa_{2_1}S_2(t)R(t) - \phi(F_1 - T_{W_1})\kappa_{2_1}S_2(t)W_1(t),\\
\frac{dP_1(t)}{dt} &= -(\kappa_{1_2} + \kappa_{1_3})P_1(t) + \kappa_{1_1}S_1(t)R(t),\\
\frac{dp_2(t)}{dt} &= -(\kappa_{2_2} + \kappa_{2_3})P_2(t) + \kappa_{2_1}S_2(t)R(t),\\
\frac{dW_1(t)}{dt} &= \kappa_{1_3}P_1(t) - \phi(F_1 - T_{W_1})\kappa_{2_1}S_2(t)W_1(t),\\
\frac{dW_2(t)}{dt} &= \kappa_{2_3}P_2(t) - \phi(F_2 - T_{W_2})\kappa_{1_1}S_1(t)W_2(t),\\
\frac{dR(t)}{dt} &= -\kappa_{1_1}S_1(t)R(t) - \kappa_{2_1}S_2(t)R(t).
\end{aligned}
\tag{48}
$$

Optimal threshold parameters were estimated as before using the FME R library, and five starting cell ratios of *P. veronii : P. putida* of 1:100, 1:10, 1:1, 1:10 and 1:100 were simulated for growth.

For the case of reciprocal inhibition, this led to extinction of *P. veronii* in three of the five starting cell ratios, and a total overestimation of its stationary phase abundances in the two other starting ratios (100:1 and 10:1) (Supplementary Fig. S3A). In addition, the total co-culture biomass for all starting cell ratios was far below the actual measured biomass (Supplementary Fig. S3B). This, therefore, indicated that reciprocal inhibition is not an accurate description of the succinate co-culture interactions. The scenario with a unilateral inhibition of *P. putida* growth by *P. veronii* byproducts, slightly improved the competitive observed outcomes, but also underestimated the total co-culture biomass and overestimated *P. veronii* growth at higher starting abundances (Supplementary Fig. S3A and B). Neither of the inhibition scenarios improved predictive outcomes of empirical data in comparison to the model without any cross-feeding (Supplementary Fig. S3C and D).

We therefore conclude that inhibition alone is an unlikely explanation for the competitive outcome on a single shared substrate such as succinate. If inhibitory aspects exist, they must appear in conjunction with other positive interactions, or else they will only lower the observed co-culture biomass.

Appendix H: Metropolis-Hasting algorithm

We want to solve the o.d.e. system (Eq.5 of main text Material and methods section),

$$\frac{dX}{dt} = F(X, \theta), \tag{49}$$

with a Metropolis-Hasting algorithm.

The Metropolis-Hasting algorithm seeks to estimate the conditional probability distribution of a model parameter $\theta \in \mathbb{R}^p$ that generated a set of empirical observations $\mathcal{O}$. Using Bayes' theorem and the information we have from the observations generated by θ, we can obtain the posterior probability function of the model parameter,

$$f(\theta|\mathcal{O}) \propto f(\mathcal{O}|\theta) f(\theta), \tag{50}$$

where $f(\mathcal{O}|\theta)$ is the information (likelihood) of the data generated by θ, and $f(\theta)$ is the prior distribution of the model parameter. We assume the errors to be independent and identically distributed according to a normal distribution with an unknown standard deviation γ depending on the type of data. Therefore, we have the relation

$$f(\vec{y_r}|\theta, \gamma) \propto \prod_{n=1}^{N_r} \exp\left\{-\frac{(y_{r,n} - x_{r,n}(\theta))^2}{2\gamma^2}\right\}. \tag{51}$$

Actually, $f(\vec{y_r}|\theta, \gamma)$ is the likelihood of the errors given the parameter θ and the unknown standard deviation γ.

Therefore, we include γ in the set of parameters to be estimated, meaning we seek to estimate $\mathbf{\Theta} = (\theta, \gamma) \in \mathbb{R}^{p+1}$. Then, we have the relations

$$f(\vec{y}|\mathbf{\Theta}) \propto \prod_{r=1}^{R} \prod_{n=1}^{N_r} \exp\left\{-\frac{(y_{r,n} - x_{r,n}(\theta))^2}{2\gamma^2}\right\}, \tag{52}$$

The algorithm seeks to find the parameters that maximize the probability $\mathbb{P}[\mathbf{\Theta}^*|\mathcal{O}]$. It uses the ratio,

$$q = \frac{f(\vec{y}|\tilde{\mathbf{\Theta}}^i)}{f(\vec{y}|\mathbf{\Theta}^{i-1})} \frac{f(\tilde{\mathbf{\Theta}}^i)}{f(\mathbf{\Theta}^{i-1})},$$

where $\mathbf{\Theta}^{i-1}$ is the value of the parameter at the $(i-1)$-th step and $\tilde{\mathbf{\Theta}}^i$ is the candidate for the i-th step.

In the special case where the prior distribution is chosen to be uniform, the second fraction vanishes.

Initial values for the model parameters $\mathbf{\Theta}^0$ can be chosen using a linear programming method minimizing minus the log-likelihood function or using biological presumption.

Algorithm 1: Algorithm 1

```
Result: Return samples set for Θ
Choose a large integer M as sample size;
Choose initial candidates for Θ^0 and φ^0 > 0;
Define a sequence of positive adaptation step sizes (η_i)_{i≥2} decaying to zero;
Choose a mean acceptance rate α* ∈ (0,1);
Set i = 1;
while i < M do
    Generate a candidate Θ̃^i = Θ^{i-1} + φ^{i-1} W_i, where W_i ~ N(0, I);
    Solve (49) for the candidate Θ̃^i, and use the resulting sequence x_n(θ̃^i) for
      computing the likelihoods as given in (52);
    Compute the ratio q = [f(y⃗|Θ̃^i) / f(y⃗|Θ^{i-1})] [f(Θ̃^i) / f(Θ^{i-1})];
    Generate a random value α ~ unif(0,1);
    if α > q then
        Θ^i = Θ^{i-1};
    else
        Θ^i = Θ̃^i;
    end
    Update φ^i using, log φ^i = log φ^{i-1} + η_i(q − α*);
    i = i + 1;
end
```

References

1. Momeni B, Xie L, Shou W. Lotka-Volterra pairwise modeling fails to capture diverse pairwise microbial interactions. eLife. 2017;6.
2. Goldford J, Lu N, Bajic D, Estrela S, Tikhonov M, Sanchez-Gorostiaga A, et al. Emergent simplicity in microbial community assembly. Science. 2018; p. 469–474.
3. van den Berg NI, Machado D, Santos S, Rocha I, Chacón J, Harcombe W, et al. Ecological modelling approaches for predicting emergent properties in microbial communities. Nature Ecology & Evolution. 2022; p. 1–11.
4. Chellaboina V, Bhat S, Haddad W, Bernstein D. Modeling and Analysis of Mass-Action Kinetics: Nonnegativity, Realizability, Reducibility, and Semistability. IEEE Control Systems Magazine. 2009;.
5. Strauss A, Yorke J. On asymptotically Autonomous Differential Equations. Mathematical Systems Theory; p. 175–182.
6. Marsland III R, Cui W, Goldford J, Sanchez A, Mehta P. Available energy flux drive a transition in the diversity, stability, and functional structure of microbial communities. PLOS Comput Biol. 2019;.
7. Biggs N. Algebraic potential theory on graphs. Bull London Math Soc. 1997;29:641–682.
