# Supplementary material for: Regulated bacterial interaction networks: A mathematical framework to describe competitive growth under inclusion of metabolite cross-feeding
Source: PLoS Comput Biol. 2023 Aug 21;19(8):e1011402. doi: 10.1371/journal.pcbi.1011402 (PMC10470959; doi:10.1371/journal.pcbi.1011402)
Supplement: Attachment — Submitted filename: Review___PCOMPBIOL_D_23_00220.pdf [file pcbi.1011402.s013.pdf]

Regulated bacterial interaction networks: A mathematical framework to describe competitive growth under inclusion of metabolite cross-feeding. (PCOMPBIOL-D-23-00220)

by Isaline Guex, Christian Mazza, Manupriyam Dubey, Maxime Batsch, Renyi Li, Jan Roelof van der Meer

The authors investigated the potential of cross-feeding interactions between two bacterial species in the manuscript. They formulate mathematical models based on chemical reaction network theory to better understand the gap between mono-culture growth kinetics and community growth through experimental research and chemical reaction networks and consider emerging biological interlinkage. Their experimental results show interesting cross-feeding interactions, which can improve the mathematical modelling of the growth kinetics of two groups of bacteria with different physiological properties. Model formulations presented in this work are reasonable, but some issues should be addressed. Still, the experimental results and mathematical approach have a good application value; it will help to understand how bacterial communities may support each other even if specific resources are utilized. However, some revisions are requried before publication in PLOS Computational Biology.

1. The maximum bacterial growth rate suggests being dependent on the initial resources and not a constant. Example: (Supp. Material) Line 36 on Page 4. Please address this.

2. The bacterial growth rate is usually considered a function of resources over time, as an example:

$$\mu(S) = \mu_{max} \frac{S}{k + S},$$

   where S is the resource, μ_{max} is the bacterial maximum growth rate and k is the half-saturation constant. This formulation is named Monod's model, which relates the maximum growth rate with resource availability. It creates a natural confusion on your model titles since you are not using this formulation. Please ensure not to create terminology conflicts with existing models in mathematical biology.

3. There are many typos in the supplementary material (example: Line 26 $F(t) = \frac{\kappa_2}{\kappa_s} X(t)$. Please review the manuscript for other typos. On equation (10), μ_{max}^2 can be confused with $(\mu_{max})^2$. I highly suggest changing the notation.

4. For the Hill function, which are the recommended values for ε? Please provide some guidelines.

5. Line 219 - 220. The decrease of microbial biomass for fast-growth bacteria after exponential growth is arguably a relevant phenomenon. In co-cultures, for example, the decrease of biomass of fast-growth bacteria may increase slowly in low-growth bacteria. The models in this manuscript do not consider the bacterial death rate, respiration rate, or maintenance, which could potentially explain this phenomenon that must not be discarded simply as an optical thing. Please provide more explanation of this situation for your experiments and your models.

6. Line 358 - 359. This is arguably not true, you must provide plenty of justification for this hypothesis in such affirmation. For example, some byproducts, like extracellular enzymes, can be consumed by bacteria that they did not produce in the first place. Furthermore, in Figure 7-B Page 16, it is very relevant the spike of *P.putida* when the initial ratio (Pve:Ppu) was considered to 100:1. You can see that the consumption of resources from slow-growth bacteria affects the growth of fast-growth bacteria around $t = 20hrs$ dramatically. In Figure 7A slow-growth, the experiment shows that *P. veronii* bacteria are able to consume putrescine at a slow rate compared to *P.putida*. Still, the byproducts produced may be consumed by the latter. Please address the further discussion of this situation which seems very relevant.
