# Supplementary material for: Regulated bacterial interaction networks: A mathematical framework to describe competitive growth under inclusion of metabolite cross-feeding
Source: PLoS Comput Biol. 2023 Aug 21;19(8):e1011402. doi: 10.1371/journal.pcbi.1011402 (PMC10470959; doi:10.1371/journal.pcbi.1011402)
Supplement: Attachment — Submitted filename: Replies_to_reviewers.pdf [file pcbi.1011402.s014.pdf]

Replies to reviewers' suggestions

Isaline Guex[1], Christian Mazza*[1], Manupriyam Dubey [2], Maxime Batsch [2], Renyi Li [2], Jan Roelof van der Meer*[2]

June 2023

1 Reviewer 1

1. **Question 1 :** *In this manuscript, Guex et al. developed a metabolite-based cross-feeding model and used it to explain the population dynamics of a two-species coculture system. By evaluating different model assumptions, they found that a concentration-dependent threshold function for byproduct utilization can best explain the observed data. They concluded that cross-feeding enables minority species to proliferate better when cross-feeding is allowed, compared to cross-feeding-free models. First, the description of model development in this manuscript appears unnecessarily lengthy. The first section of Results (section 3.1) are technical, and it would be helpful to move these details to the Methods or Supplementary Material. I appreciate the authors' approach of introducing their model in a stepwise manner, starting from a single species model all the way up to a 2-species model with regulated cross-feeding. However, most of these models have been established. Therefore, a concise summary of the key differences between the authors' model and past models would suffice for readers to understand the innovation.*
   Answer 1 : We thank the reviewer for the general appreciation. We have shortened the technical description of the model description here and have moved its full mathematical development to Supplementary text, Appendices S1-S7.
   Action Plan 1 : The text part on pages 4-7 was condensed to 2 pages in the revised manuscript, and the mathematics background was moved to Appendices S1-S7.

2. **Question 2 :** *Following my previous comment, the model description in the Methods section (5.2) is not helpful too. Introducing the basic concepts of CRN models, mass action kinetics, and generalized consumer resource models seems to be unnecessary, as they are quite standard. If the authors still want to include them, the Supplementary Material would be a better place. Instead, a clear description of the equations used in the CURRENT study is expected in the Methods section. Although the authors provide an example in Section 5.2.3, this is poorly explained. For example, the waste*

concentration F3 is undefined since there are only two waste products in CRN. It is also unclear whether κ_i and $\tilde{\kappa}_i$ are fundamental parameters or functions of other fundamental parameters. Furthermore, it is not clear whether the example was a generalized consumer-resource model or a model developed by this study.

Answer 2 : We thank the reviewer for this comment. As mentioned above, we have moved most of the mathematics background and development to the Supplementary text. Paged 18-20 of the old text were shortened, to leave only the relevant description of the RBN with cross-feeding.

The kappa are fundamental parameters, corresponding to reaction rates in the RBN. See, for example, Appendix S6.

F3 was defined as the resource concentration, but this was changed to R for clarity.

The difference to a generalized consumer resource model is explained in Appendix S5. Indeed, the RBN model was specifically developed for this study.

Action Plan 2 : We have shortened this part of the Methods section to emphasize the equations used in the study, and less so, their development from basic principles. A full mathematical development was moved to Supplementary text, as suggested.

3. **Question 3 :** *Based on the explanation provided, I am concerned that the model presented in this study is almost identical to, and even simpler (e.g., no metabolite-dependent growth inhibition) than, the model developed in Liao et al. PLoS Comput Biol, 2020. Both are kinetic, metabolite-based cross-feeding models, with the potential difference being that the current study used two activation functions to simulate the uptake rate of metabolic byproducts. However, the underlying math of the two functions does not make sense to me. The authors called Eq. 6 "permanent cross-feeding" and Eq. 9 "regulatory cross-feeding". For Eq. 6, the reaction rate under mass action kinetics would be $\kappa_1 4 * [S_1] * [W_2]$, which depends on the byproduct concentration $[W_2]$. Since this term goes to zero when $[W_2]$ equals zero, the cross-feeding rate it quantifies cannot be permanent.*

   Answer 3 : We thank the reviewer for this comment and apologize for the omission to directly include the Liao et study of 2020. Our model is not a repetition of the Liao study, but was developed from a conceptual CRN network on a mathematical basis, and also in close conjunction to the experimental results ; hence, growth-inhibition terms were not specifically generalized in the model, because they were not relevant for the experiments. We did however, for the revisions, include simulations with growth inhibitory terms, which result in predictions very far from what was empirically observed. Hence, we don't believe this a useful addition to the work here.

   What we mean with "permanent cross-feeding" in Eq. 6 refers to the situation where cross-feeding is allowed at any concentration of the by-

product $[W_2]$. (Indeed, this implies that when the byproduct concentration goes to zero, the rate becomes zero as well.) In contrast, with Eq.9 we refer to a situation where the rate is dependent on a concentration threshold, above which it appears (in a concentration-dependent manner). Biologically speaking this is an important difference, because in the first case one would allow an infinitely sensitive uptake reaction by the cells, whereas in the other one would account for an uptake reaction to be thermodynamically limited.

Action plan 3 : We revisited the description of Eq. 6 and Eq. 9 on page 6 in the new Equation 3 of the revised text. The other details were described in Appendix 5 and 6, which prove the statement that constant utilization of appearing byproducts is unlikely.
The differences to the Liao model are explained in l. 111-117 ; l. 180-182, l. 420-437 of the revised text.

4. **Question 4 :** *Moreover, the mass action kinetics of Eq. 6 (i.e.,* $\kappa_1 4 * [S_1] * [W_2]$*) already accounts for the concentration-dependent byproduct uptake. Why did the authors introduce another concentration-dependent functions (Eq. 9 and 10) that models the same mechanism ? I would assume the reaction rate of Eq. 9 is* $\phi(F_2 - T_w 1) * \kappa_1 4 * [S_1] * [W_2] (= F_2)$*. If this is correct, both* $\phi(F_2 - T_w 1)$ *and* F_2 *quantify the concentration-dependent effect. At the minimum, the authors need to compare their model with the one presented in Liao et al and justify why they introduced two additional regulatory functions given that the concentration-dependency of byproduct uptake can be modeled by mass action kinetics of Eq. 6.*

   Answer 4 : As explained above to point 3 it is not so much the byproduct concentration dependency that counts, but its non-linearity and the inclusion of the threshold. In any case, including non-linearity shows better simulation results than a 'permanent' linear byproduct consumption. There is no 'double' inclusion of the concentration-dependent waste uptake. See, e.g., Eq. 42-44 in Appendix S6 of the (final) RBN model. The indicative function ϕ is imposed on the utilization of the waste concentration for biomass formation.

   Action Plan 4 : No further action needed. See the Action Plan to Question 3 and further Appendix S6.

5. **Question 5 :** *Aside from the mathematical issues, my other concern is with the conclusion that cross-feeding is responsible for the difference between the observed and simulated species ratios in a "cross-feeding-free" model. While cross-feeding is a possible explanation for this difference, many other mechanisms can be explanatory too, especially considering that the model-experiment difference is generally small (though significant) across all experimental settings (Fig. 4C). For example, the model did not account for the inhibitory effects of byproduct wastes or the proportion of wastes that could be utilized by the other microbe (all wastes were combined into a pseudo molecule).*

   Answer 5 : We thank the reviewer for this comment. Indeed, we lump

for simplicity all potential appearing metabolites into one sum. This facilitates calculating the share of cross-feeding to yield gains and losses. As to the question of including inhibitory reactions, we now included simulations that would explain their effects. However, these simulations predict that inhibition will lead to (much) smaller stationary phase biomass abundances and underestimation of *P. veronii* proportions, which is not what we observed empirically. Therefore, we conclude that the interaction (in the case of *P. veronii* and *P. putida*) must occur primarily through metabolic cross-feeding.
Action Plan 5 : To show this more clearly, we repeated model simulations with five different starting cell ratios of both species and either reciprocal or unilateral inhibition, as suggested by the reviewer. This result is described in l. 295-299 of the revised manuscript. The corresponding model is presented in Appendix S7 and the results in SI Fig. 3.

6. **Question 6 :** *Is it feasible for the authors to validate the presence of cross-feeding and determine the specific metabolites that mediate this positive interaction ?*
Answer 6 : We appreciate this suggestion and had reflected about this ourselves, already. Previous exometabolomics data (Hadadi et al., 2020) on one of the used bacterial strains had already indicated that several dozens of non-primary substrate compounds appear in exponentially growing cultures, which is in agreement with typical results in the literature. Therefore, an experiment to 'cross-wise' grow either strain on spent medium of the other, would likely show some net growth. However, given that exponential growth is a dynamic situation (as simulated in Figure 5 in the manuscript), we have not found a suitable experimental condition which would mimic exactly the conditions during growth but without the primary substrate being present. (One would have to grow the strains in chemostats and monitor short growth-bursts on addition of presumed byproducts, but we don't have this capacity in our laboratory). Therefore, we are afraid we cannot satisfy this suggestion, except by arguing for its likelihood on the basis of literature data, and providing validation in terms of estimating the yield proportion from metabolite cross-feeding.
7. **Question 7 :** *Other specific comments : The authors used "positive feedback loop" in a couple of places. Cross-feeding is for sure a positive interaction (a -> b) but I am not sure if it forms an overall loop (a -> b -> a). The notations used in Eq. 9 are a bit confusing. Why does the uptake of byproduct W_2 have a threshold of T_{w1} while the uptake of byproduct W_1 has a threshold of T_{w2} ? Is this a typo in the subscripts ?*
Answer 7 : We speak here of feedback 'loop' here, to reflect its dynamic occurrence during growth (e.g., metabolism releases W1, which is used by species 2 for growth). However, we agree that using 'feedback' only would be sufficient.
Action plan 7 We have revisited the text to avoid using the phrase

'feedback loop' and have changed the notation of the threshold names. We have verified and corrected the notations of the reciprocal waste utilization.

8. **Question 8 :** *A parenthesis is missing somewhere between line 238-241 Line 283-284 : could not find model B of equation 7*
   Answer and Action plan 8 : We apologize for this typographical error, which we have corrected.
   The model of Figure 5B corresponds to the ODE's system of equation (44) in Appendix S6 with $\phi \equiv 1$. With constant rates of cross-feeding, the stationary byproduct concentration is zero, which doesn't correspond to experimental observations. The mathematical proof is given in Appendix S4. We added this information to line 290-293 of the main text.

2 Reviewer 2

The authors investigated the potential of cross-feeding interactions between two bacterial species in the manuscript. They formulate mathematical models based on chemical reaction network theory to better understand the gap between mono-culture growth kinetics and community growth through experimental research and chemical reaction networks and consider emerging bio-logical interlinkage. Their experimental results show interesting cross-feeding interactions, which can improve the mathematical modelling of the growth kinetics of two groups of bacteria with different physiological properties. Model formulations presented in this work are reasonable, but some issues should be addressed. Still, the experimental results and mathematical approach have a good application value ; it will help to understand how bacterial communities may support each other even if specific resources are utilized. However, some revisions are required before publication in PLOS Computational Biology.
Reply : We thank the reviewer for the general remarks on the work.

1. **Question 1 :** *The maximum bacterial growth rate suggests being dependent on the initial resources and not a constant. Example : (Supp. Material) Line 36 on Page 4. Please address this.*
   Answer 1 : We thank the reviewer for this remark. The population growth rate is dependent on the resource concentration, but the maximum growth rate (mu-max) is a constant. However, the observed mu will be different depending on the primary substrate and even on the initial concentrations of that substrate, which results in the typical Monod (Ks) dependency. With our fitting method (Monte-Carlo described in Appendix S8), we estimated the intrinsic growth rates for each of the culture conditions.
   Action Plan 1 : We have revisited the text to ensure that these statements are correct here.
2. **Question 2 :** *The bacterial growth rate is usually considered a function*

of resources over time, as an example :

$$\mu(S) = \mu_{max} \frac{S}{k + S},$$

where S is the resource, μ_{max} is the bacterial maximum growth rate and k is the half-saturation constant. This formulation is named Monod's model, which relates the maximum growth rate with resource availability. It creates a natural confusion on your model titles since you are not using this formulation. Please ensure not to create terminology conflicts with existing models in mathematical biology.

Answer 2 : We thank the reviewer for this remark. It was not our goal to create terminology conflicts, but to provide a mathematical background for the development of the (empirical) Monod equation from chemical reaction networks (Appendix S2). Essentially, the equation shown in Eq. 18 of that appendix is the Monod equation. The importance of using slightly different variable names is that it makes it easier to describe coculture dynamics, because the original Monod equation does not take byproducts (formation and consumption) into account.

Action Plan 2 : We included a new text on line 105-107 and Appendix S2 to clarify this aspect better.

3. **Question 3 :** *There are many typos in the supplementary material (example : Line 26 $F(t) = \frac{\kappa_2}{\kappa_3} X(t)$. Please review the manuscript for other typos. On equation (10), μ^2_{max} can be confused with $(\mu_{max})^2$. I highly suggest changing the notation.*

   Answer 3 : We apologize for any possible typographical errors and have done our best to go again through any listed equation. In this particular case, the superscript referred to the species number.

   Action Plan 3 : We revisited and verified all the equations (particularly also in the Appendices), and checked superscripts that can be potentially mistaken. The μ^i_{max} was changed to $\mu^{(i)}_{max}$ to indicate the species designation.

4. **Question 4 :** *For the Hill function, which are the recommended values for ϵ ? Please provide some guidelines.*

   Answer and Action Plan 4 : We thank the reviewer for the question. We have provided a new supplementary figure S3 with examples of Hill functions for several values of ϵ. One can see that at an ϵ of 100, the value is close to the indicative function, what corresponds to a high Hill factor k.

5. **Question 5 :** *Line 219 - 220. The decrease of microbial biomass for fast-growth bacteria after exponential growth is arguably a relevant phenomenon. In co-cultures, for example, the decrease of biomass of fast-growth bacteria may increase slowly in low-growth bacteria. The models in this manuscript do not consider the bacterial death rate, respiration rate, or maintenance, which could potentially explain this phenomenon that must*

not be discarded simply as an optical thing. Please provide more explanation of this situation for your experiments and your models.

Answer and Action plan 5 : We thank the reviewer for this observation and suggestion. Indeed, it is very difficult to account for all possible phenomena, particularly, those that could concern stationary phase and cell death. However, our work here primarily deals with phenomena during competition in exponentially growing co-culture populations, where cell death is limited. (As a matter of fact, we measure both culture turbidities and flow cytometry living cell counts ; the latter does account for cell death). Secondly, although cell death and lysis will potentially result in liberated nutrients, which become available for other strains, the thermodynamic conversion of such nutrients will follow the same yield considerations as primary substrates.

The 'optical' thing is the particularity here of *P. putida* to decrease culture turbidity at early stationary phase. However, this is not due to cell death, but decreasing cell size and potential cell clumping. We added this explanation to the main text on lines 341-342 and 513-514.

6. **Question 6 :** *Line 358 - 359. This is arguably not true, you must provide plenty of justification for this hypothesis in such affirmation. For example, some byproducts, like extracellular enzymes, can be consumed by bacteria that they did not produce in the first place. Furthermore, in Figure 7-B Page 16, it is very relevant the spike of P.putida when the initial ratio* ($Pve : Ppu$) *was considered to 100 :1. You can see that the consumption of resources from slow-growth bacteria affects the growth of fast-growth bacteria around* $t = 20hrs$ *dramatically. In Figure 7A slow-growth, the experiment shows that P. veronii bacteria are able to consume putrescine at a slow rate compared to P.putida. Still, the byproducts produced may be consumed by the latter. Please address the further discussion of this situation which seems very relevant.*

   Answer 6 : We thank the reviewer for this remark. The statement of line 358-359 suggested that both species in the indifference scenario indeed use both substrates independently, although we did see a small cross-feeding appear, as described in l. 371-389.

   Indeed, the reviewer is correct that an increase of *P. putida* fluorescence appears after 20 h in the co-culture with both substrates, but only when *P. veronii* is in excess. We did consider this cross-feeding, and showed its extent in Fig. 7D. The magnitude of the cross-feeding compared to the case of succinate (competition for a single same resource), however, is much smaller. Moreover, the increase is only temporarily, which could mean that it is not an increase in biomass, but only in fluorescence expression.

   Action Plan 6 : We (already) did acknowledge that there is cross-feeding arising even for the indifference scenario, but with a much smaller magnitude than in case of succinate (l. 371-389). We revisited this paragraph to ensure that it correctly reflects the considerations of the

reviewer here.
