# Supplementary material for: Regulated bacterial interaction networks: A mathematical framework to describe competitive growth under inclusion of metabolite cross-feeding
Source: PLoS Comput Biol. 2023 Aug 21;19(8):e1011402. doi: 10.1371/journal.pcbi.1011402 (PMC10470959; doi:10.1371/journal.pcbi.1011402)
Supplement: Attachment — Submitted filename: Revisited___PCOMPBIOL_D_23_00220__Response_.pdf [file pcbi.1011402.s015.pdf]

[Revisited] Regulated bacterial interaction networks: A mathematical framework to describe competitive growth under inclusion of metabolite cross-feeding. (PCOMPBIOL-D-23-00220)

by Isaline Guex, Christian Mazza, Manupriyam Dubey, Maxime Batsch, Renyi Li, Jan Roelof van der Meer

This file is regarding the revised submission provided by the authors, including the feedback provided before. The new manuscript has been published, and the structure, notation and clarity have been improved. Furthermore, additional material has been added, increasing their findings' arguments. Based on the actions taken by the authors regarding the feedback previously provided, and the new presentation of the work titled "Regulated bacterial interaction networks: A mathematical framework to describe competitive growth under inclusion of metabolite" **I will recommend the publication of this manuscript in the PLOS Computational Biology Journal.**

Comments about the feedback previously provided:

1. The maximum bacterial growth rate suggests being dependent on the initial resources and not a constant. Example: (Supp. Material) Line 36 on Page 4. Please address this.
    - *[Resolved] The authors pointed out that the maximum growth rate depends on the primary substrate and is estimated using the Monte-Carlo method.*
2. The bacterial growth rate is usually considered a function of resources over time, as an example:

$$\mu(S) = \mu_{max} \frac{S}{k + S},$$

where S is the resource, μ_{max} is the bacterial maximum growth rate and k is the half-saturation constant. This formulation is named Monod's model, which relates the maximum growth rate with resource availability.

It creates a natural confusion on your model titles since you are not using this formulation. Please ensure not to create terminology conflicts with existing models in mathematical biology.

- *[Resolved] The authors clarified that the saturation function they have formulated has the same structure as Monod's model and renamed it as Monod-model of substrate to biomass to avoid confusion.*

3. There are many typos in the supplementary material (example: Line 26 $F(t) = \frac{\kappa_2}{\kappa_s} X(t)$. Please review the manuscript for other typos. On equation (10), μ^2_{max} can be confused with $(\mu_{max})^2$. I highly suggest changing the notation.

   - *[Resolved] The authors corrected the typos and updated their notation.*

4. For the Hill function, which are the recommended values for ε? Please provide some guidelines.

   - *[Resolved] The authors provided a figure of Hill's function FigS2.*

5. Line 219 - 220. The decrease of microbial biomass for fast-growth bacteria after exponential growth is arguably a relevant phenomenon. In co-cultures, for example, the decrease of biomass of fast-growth bacteria may increase slowly in low-growth bacteria. The models in this manuscript do not consider the bacterial death rate, respiration rate, or maintenance, which could potentially explain this phenomenon that must not be discarded simply as an optical thing. Please provide more explanation of this situation for your experiments and your models.

   - *[Resolved] The authors provided a justification for the phenomenon in the text.*

6. Line 358 - 359. This is arguably not true, you must provide plenty of justification for this hypothesis in such affirmation. For example, some byproducts, like extracellular enzymes, can be consumed by bacteria that they did not produce in the first place. Furthermore, in Figure 7-B Page 16, it is very relevant the spike of *P.putida* when the initial ratio (Pve:Ppu) was considered to 100:1. You can see that the consumption of resources from slow-growth bacteria affects the growth of fast-growth bacteria around $t = 20hrs$ dramatically. In Figure 7A slow-growth, the experiment shows that *P. veronii* bacteria are able to consume putrescine at a slow rate compared to *P.putida*. Still, the byproducts produced may be consumed by the latter. Please address the further discussion of this situation which seems very relevant.

   - *[Resolved] The authors provided a revisited the paragraph.*
